# PD1^Hi^ CD8^+^ T cells correlate with exhausted signature and poor clinical outcome in hepatocellular carcinoma

**DOI:** 10.1186/s40425-019-0814-7

**Published:** 2019-11-29

**Authors:** Jiaqiang Ma, Bohao Zheng, Shyamal Goswami, Lu Meng, Dandan Zhang, Chunmei Cao, Teng Li, Fangming Zhu, Lijie Ma, Zhao Zhang, Shuhao Zhang, Meng Duan, Qin Chen, Qiang Gao, Xiaoming Zhang

**Affiliations:** 1 The Center for Microbes, Development and Health, Key Laboratory of Molecular Virology & Immunology, Institut Pasteur of Shanghai, Chinese Academy of Sciences/University of Chinese Academy of Sciences, Shanghai, 200031 China; 2 Department of Liver Surgery and Transplantation, and Key Laboratory of Carcinogenesis and Cancer Invasion (Ministry of Education), Liver Cancer Institute, Zhongshan Hospital, Fudan University, 180 Fenglin Road, Shanghai, 200032 China; 3 Shanghai Key Laboratory of Bio-Energy Crops, School of Life Sciences, Shanghai University, Shanghai, 200444 People’s Republic of China; 4 MOE Key Laboratory of Metabolism and Molecular Medicine, Department of Biochemistry and Molecular Biology, School of Basic Medical Sciences, Fudan University, Shanghai, 200032 China; 5 Cancer Institute, Fudan University Shanghai Cancer Center, Shanghai, 200032 China

**Keywords:** Hepatocellular carcinoma, CD8^+^ T cells, T cell exhaustion, Multiplex immunohistochemistry, Spatial analysis

## Abstract

**Background:**

CD8^+^ T cells differentiate into exhausted status within tumors, including hepatocellular carcinoma (HCC), which constitutes a solid barrier to effective anti-tumor immunity. A detailed characterization of exhausted T cells and their prognostic value in HCC is lacking.

**Methods:**

We collected fresh tumor tissues with adjacent non-tumor liver tissues and blood specimens of 56 HCC patients, as well as archived samples from two independent cohorts of HCC patients (*n* = 358 and *n* = 254), who underwent surgical resection. Flow cytometry and multiplex immunostaining were used to characterize CD8^+^ T cells. Patient prognosis was evaluated by Kaplan-Meier analysis and Cox regression analysis.

**Results:**

CD8^+^ T cells were classified into three distinct subpopulations: PD1^Hi^, PD1^Int^ and PD1^−^. PD1^Hi^ CD8^+^ T cells were significantly enriched in tumor compared to adjacent non-tumor liver tissues. PD1^Hi^ CD8^+^ T cells highly expressed exhaustion-related inhibitory receptors (TIM3, CTLA-4, etc.) and transcription factors (Eomes, BATF, etc.). In addition, PD1^Hi^ CD8^+^ T cells expressed low levels of cytotoxic molecules and displayed a compromised capacity to produce pro-inflammatory cytokines while the expression of anti-inflammatory IL-10 was up-regulated following mitotic stimulation. Furthermore, PD1^Hi^ CD8^+^ T cells shared features with tissue resident memory T cells and were also characterized in an aberrantly activated status with an apoptosis-prone potential. In two independent cohorts of HCC patients (*n* = 358 and *n* = 254), we demonstrated that PD1^Hi^ or TIM3^+^PD1^Hi^ CD8^+^ T cells were significantly correlated with poor prognosis, and the latter was positioned in close proximity to PD-L1^+^ tumor associated macrophages.

**Conclusion:**

The current study unveils the unique features of PD1^Hi^ CD8^+^ exhausted T cells in HCC, and also suggests that exhausted T cells could act as a biomarker to select the most care-demanding patients for tailored therapies.

## Introduction

Hepatocellular carcinoma (HCC) is the most prevalent primary liver cancer and a major cause of cancer mortality. This malignancy usually develops on chronic inflammatory liver disease (e.g. fibrosis or cirrhosis) and correlates with certain risk factors such as hepatitis B virus (HBV), hepatitis C virus, alcohol abuse and metabolic diseases [1, 2]. The potentially curative therapies including surgical resection, liver transplantation and radiofrequency ablation are only suitable to patients in early stages, while the majority of advanced HCC patients are with limited therapeutic options [3].

Cancer immunotherapies have dramatically altered the oncological treatment landscape during the past decade [1]. For HCC, various studies have shown that shifting of local immune responses toward an anti-tumor direction, like increased infiltrations of cytotoxic T, NK, and NKT cells, are positive prognostic factors, highlighting the potentials of immunotherapy in HCC management [4, 5]. Recent success of immune checkpoint inhibitors (ICIs) such as Nivolumab and Pembrolizumab by blocking the programmed cell death 1 (PD-1)-PD-L1 pathway revokes the investigations on immune therapy in HCC [6]. However, the overall response rate of checkpoint inhibitors is only 15–20% in HCC patients, indicating the urgent needs to overcome this obstruction of low response rate thorough evaluation of the mechanisms of local and systemic anti-tumor immune responses in HCC [6].

Cytotoxic CD8^+^ T cells play a critical role in anti-tumor immunity. However, in the context of suppressive tumor microenvironment and prolonged antigen exposure, tumor-specific effector CD8^+^ T cells are prone to differentiate into a stage called “T cell exhaustion”. Such exhausted CD8^+^ T cells are distinct from functional effector and memory T cells manifested by hierarchical loss the ability of cytokine production (IL2, TNF-α and IFN-γ) and killing capacity [7]. Exhausted CD8^+^ T cells are characterized by a distinct transcriptional programs (such as low expression of T-bet and TCF1 and high expression of Eomes and TOX) [7, 8] and a proliferative status [9]. Of note, overexpression of multiple inhibitory receptors (such as PD-1, lymphocyte-activation gene 3 (LAG3), T cell immunoglobulin domain and mucin domain-containing protein 3 (TIM-3; also known as HAVCR2) and cytotoxic T lymphocyte-associated antigen-4 (CTLA4) was commonly observed in exhausted CD8^+^ T cells and the intensity and number of immune inhibitory receptors expressed by exhausted T cells positively correlate with severity of exhaustion [10, 11]. Consistently, increased expression of PD1, TIM3, LAG3 and CTLA4 on CD8^+^ T cells were observed in HCC [12–15]. However, the functional role and clinical significance of the heterogeneous expression of immune inhibitory receptors in HCC-infiltrating CD8^+^ T cells remain largely unknown.

To address these issues, we characterized the PD1 and TIM3 expression in HCC-infiltrating CD8^+^ T cells using high-throughput flow cytometry and multiplex immunohistochemistry. By investigating their phenotype, function and clinical impacts in HCC patients, we offer novel insights into PD1 heterogeneous expression signature and spatial interactions with PD-L1^+^ macrophages in tumor microenvironment, advancing our understanding of T cell exhaustion and raising potential therapeutic opportunity for HCC.

## Methods

### Patients and tissue microarray (TMA) construction

Fresh paired HCC tissues and peripheral blood samples were obtained from 56 patients receiving hepatectomy at Zhongshan Hospital of Fudan University (Shanghai, China) from Jan. to Sep. 2015. TMAs were constructed using specimens from two independent cohorts of HCC patients (*n* = 358 and *n* = 254) who underwent primary resection in 2006 and 2007 respectively. Tissue cores of 1 mm diameter were selected from high immune cells infiltrated region in paraffin-embedded tumor and peri-tumor tissue based on the HE staining. Sections (4 μm) were cut and applied to APES- coated slides as previously described [4]. Patient informed consent was obtained and the study was approved by the institutional ethics committee. Tumor differentiation was graded by the Edmondson grading system [4]. None of the patients received antitumor or immunosuppressive treatments before surgery.

### Multiplex immunohistochemistry and quantitative analysis

Multiplex immunohistochemistry (mIHC) was performed according to manufacturer’s instruction (PerkinElmer, Opal® Kit). The process was performed on the following antibodies and fluorescent dyes in the flowing order: CD3/Opal570, CD8/Opal690, PD1/Opal520, TIM3/Opal650. Detailed procedures of mIHC and quantitative analysis are presented in Additional file 1. Slides were scanned and imaged using the PerkinElmer Vectra3® platform and were analyzed in batches using PerkinElmer inform and R script for quantification of positively stained cells.

### Isolation of mononuclear cells from peripheral blood and tissues

Peripheral mononuclear leukocytes were isolated by Lymphoprep (STEMCELL Technologies) density gradient centrifugation, and fresh tissue-infiltrating mononuclear leukocytes were obtained as described previously [16]. Detailed procedures of isolation of mononuclear cells are presented in Additional file 1.

### Flow cytometry

Peripheral blood leukocytes as well as tumor- and adjacent non-tumor liver tissue-infiltrating leukocytes were stained with fluorochrome-conjugated antibodies against CD3, CD4, CD8, PD1, and TIM3, or control antibodies to identify the exhausted T cells in a flow cytometry analyzer (BD LSR Fortessa, BD, CA). Data were analyzed by FlowJo software (v9.3.2; TreeStar, USA). PD1-positivity among CD8^+^ T cells was defined based on isotype antibody control and the separation of PD1-high (PD1^Hi^) from PD1-intermediate (PD1^int^) among PD1^+^ CD8^+^T cells was based on mean fluorescence intensity (MFI) and the expression of TIM 3 [17, 18]. Details of the fluorochrome-conjugated antibodies and isotype controls are listed in Additional file 2: Table S1.

### Statistical analysis

Statistical analysis was performed with the R software, SPSS (v22, IBM, Armonk, NY) and Prism 6.0 (GraphPad Soft Inc., SanDiego, CA) softwares. Comparisons were performed using Student’s *t*-test, ANOVA test or Chi-square test as appropriate. Kaplan–Meier curves were used and estimated by the log-rank test. Patients were classified as ‘low’ and ‘high’ groups according to the Youden index to achieve the optimal cutoffs. The number of nearest neighbor was calculated on the assigned coordinates of each cell and performed by R software using the *spatstat* package. Multivariate analysis was performed by Cox regression analysis. Two-sided *P* <  0.05 considered as statistically significant.

## Results

### Increased frequency of PD1^Hi^ CD8^+^ T cells in the tumor tissues of HCC patients

**Fig. 1:**
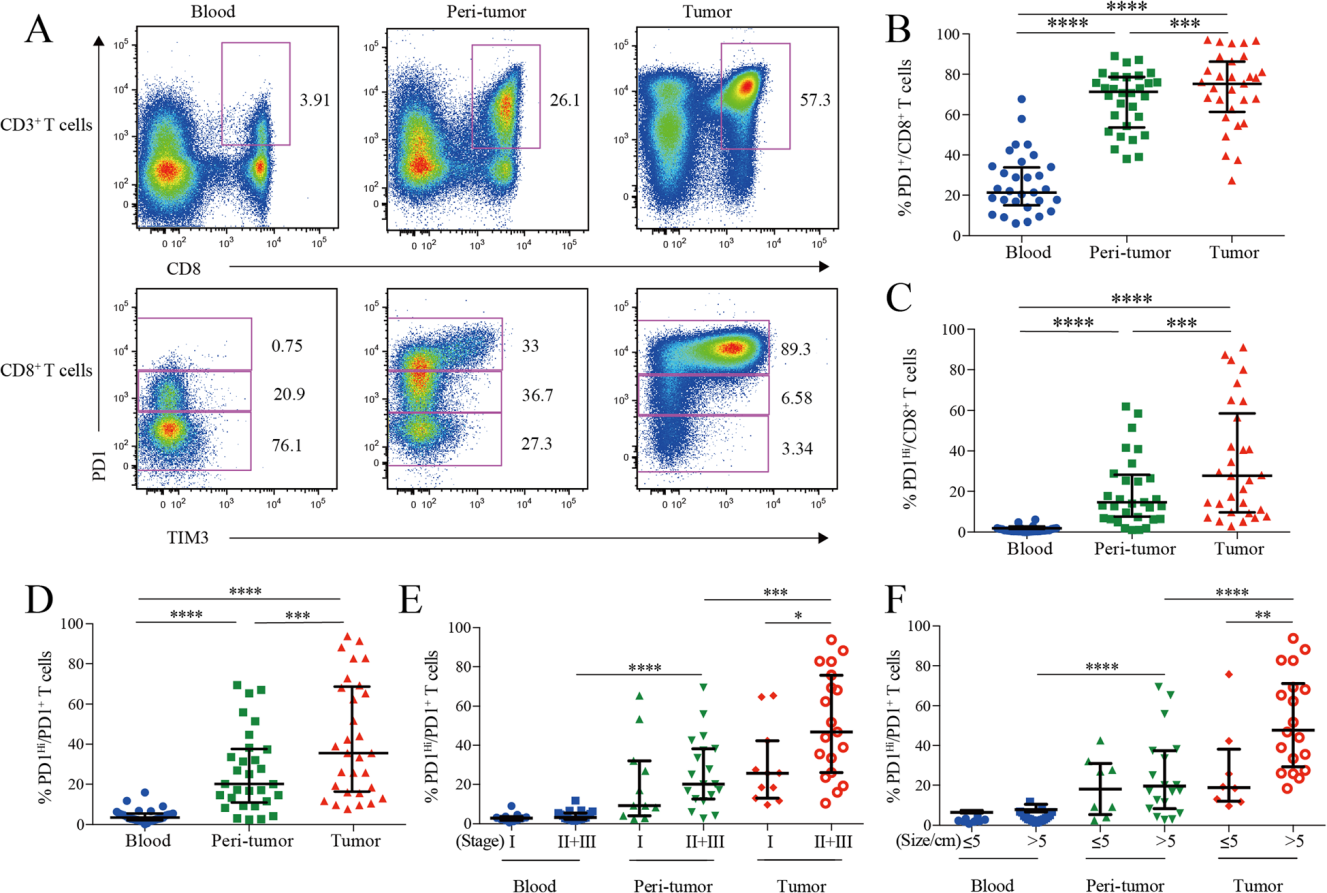
PD1 and TIM3 expression on HCC infiltrating CD8^+^ cytotoxic T cells. **a**, Representative flow cytometric plots to show the expression of PD1 and TIM3 on CD8^+^ cytotoxic T cells from paired blood, peri-tumor and tumor tissues of HCC patients. **b-d**, Comparison of the frequencies of PD1^+^ among CD8^+^ T cells (**b**), PD1^Hi^ among CD8^+^ T cells (**c**) and PD1^Hi^ among CD8^+^PD1^+^ T cells (**d**) across paired blood, peri-tumor and tumor tissues of HCC patients (*n* = 30). **e-f**, Comparison of the frequencies of PD1^Hi^ among CD8^+^PD1^+^ T cells in relation to tumor stages (E, 11 stage I and 19 stage II and III) and tumor size (F, 8 tumor size≤5 cm and 19 tumor size> 5 cm) across paired blood, peri-tumor and tumor tissues of 11 stage I and 19 stage II and III HCC patients; and paired blood, peri-tumor tissue and tumor tissue from 8 tumor size≤5 cm and 19 tumor size> 5 cm HCC patients. Error bars indicated median with interquartile range. Significance was assessed by Wilcoxon matched-pairs signed rank test. *, *P* < 0.05; **, *P* < 0.01; ***, *P* < 0.001; and ****, *P* < 0.0001

Previously, PD1 and TIM3 expression were found to be upregulated in tumor-infiltrating lymphocytes (TILs) of tumor-bearing mice and HCC patients [15]. Co-expression of PD1 and TIM3 on CD8^+^ T cells was also demonstrated to be associated with T cell exhaustion in melanoma and non–small cell lung carcinoma [19, 20]. Herein, to assess the potential role of PD1 and TIM3 in HCC immunopathology, we first examined the expression of PD1 and TIM3 on CD8^+^ T cells from 30 HCC patients including paired peripheral blood, peri-tumor and tumor tissues by flow cytometry. As shown in Fig. 1a, PD1^+^ CD8^+^T cells were readily identified in blood and liver tissues. In addition, PD1^+^ cells can be further subdivided into PD1-high (PD1^Hi^) and PD1-intermediate (PD1^int^) and TIM3 expression was limited to PD1^Hi^ CD8^+^T cells. Consequently, based on the TIM3 expression, PD1^Hi^ CD8^+^ TILs could be also subdivided into TIM3^−^PD1^Hi^ and TIM3^+^PD1^Hi^ CD8^+^ TILs.

We then compared the frequencies of PD1-expressing cells from different blood and HCC tissue. PD1^+^ cells among CD8^+^ T cells in peripheral blood (median = 21.70, 15.49–34.27% [IQR, same for the following]) was greatly increased in peri-tumor tissues (median = 70.82, 54.63–78.01%; *P* <  0.0001) and further elevated in tumor tissues (median = 76.98, 67.51–88.11%; *P* <  0.0001) (Fig. 1b). The frequency of PD1^Hi^ cells among CD8^+^ T cells showed a similar trend: tumor tissues harbored a significantly higher frequency of PD1^Hi ^CD8^+^ T cells (median = 26.60, 9.60–58.55%) than that of peri-tumor CD8^+^ T cells (median = 13.47, 6.35–27.05%; *P* = 0.0006), while blood contained minimal PD1^Hi^ CD8^+^ T cells (Fig. 1c). In line with the above data, blood CD8^+^ T cells harbored the highest PD1^−^ cells (median = 75.04, 62.43–80.12%) while peri-tumor contained the highest PD1^Int^ cells (median = 47.66, 39.66–57.30%) (Additional file 3: Figure S1A and B).

We also studied the frequency of PD1^Hi^ cells among PD1^+^ CD8^+^ T cells to see the relative sharing of PD1^Hi^ cells within PD1^+^ compartment. Again, tumor tissues had the highest frequency (median = 35.54, 18.38–67.53%) (Fig. 1d), indicating an efficient transition from PD1^int^ to PD1^Hi^ of CD8^+^ T cells within tumor microenvironment. In addition, the frequency of PD1^Hi^ cells in PD1^+^ CD8^+^ T cells in advanced stage HCC patients (stages II and III, *n* = 19) was two-fold higher than those in early stage (stage I, *n* = 11; *P* = 0.026; Fig. 1e). Likewise, the frequency of PD1^Hi^ in PD1^+^ CD8^+^ T cells was also positively associated with larger tumor size (*P* = 0.008) (Fig. 1f). Moreover, the frequency of PD1^+^ and PD1^Hi^ among CD8^+^ T cells showed the similar clinical associations as the above (Additional file 3: Figure S1C-F). These results indicate that HCC progression is associated with an enrichment of PD1^Hi^ CD8^+^ T cells within tumor tissues.

### An exhausted and aberrantly differentiated phenotype of PD1^Hi^ CD8^+^ T cells in HCC

**Fig. 2:**
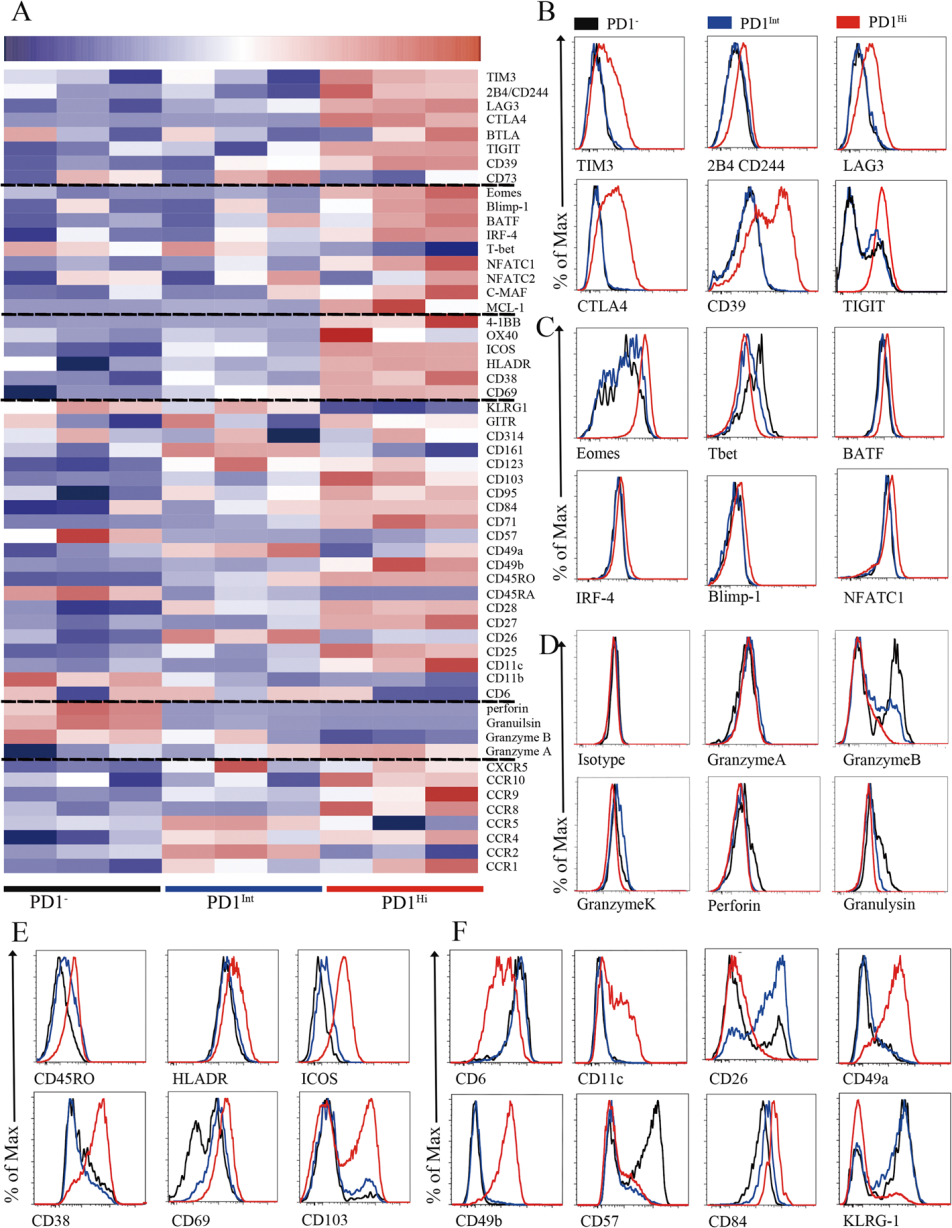
Phenotypic characteristics of tumor infiltrating PD1^Hi^ CD8^+^ T cells in HCC. **a**, A heatmap to show the global phenotypic characteristics of PD1^Hi^, PD1^Int^ and PD1^−^ CD8^+^T cells from HCC tumor tissues detected by the flow cytometry. Data represent three individual patients. **b-f,** Representative flow cytometric overlays of different markers expressed by tumor infiltrating PD1^Hi^ (red line), PD1^Int^ (blue line) and PD1^−^ (black line) CD8^+^T cells, including co-inhibitory receptors **(b)**, exhaustion associated transcription factors **(c),** cytotoxic molecules **(d),** differentiation and activation markers **(e-f)**

To better understand the phenotypic characteristics and functional status of PD1^Hi^, PD1^Int^ and PD1^−^ CD8^+^ TILs in HCC, we performed a comprehensive characterization of these cells by flow cytometry, including 64 cluster of differentiation (CD) surface markers, 24 transcription factors, 18 chemokine receptors and 10 cytokine receptors. The differentially expressed makers among PD1^Hi^, PD1^Int^, and PD1^−^ CD8^+^ TILs were displayed in the heat map (Fig. 2a**)** and selected markers were presented as overlaid histograms (Fig. 2b-f and Additional file 4: Figure S3 and Additional file 5: Figure S4).

The predominant feature of PD1^Hi^CD8^+^ TILs is that they displayed an exhausted phenotype [10, 21]. First, PD1^Hi^ CD8^+^ TILs expressed high levels of well-known inhibitory receptors: TIM3, CTLA4, 2B4(CD244), LAG3, CD39 and TIGIT (Fig. 2a and b), which were confirmed at mRNA level for most of them (Additional file 6: Figure S2). Second, PD1^Hi^ CD8^+^ TILs exhibited a transcription factor signature of exhausted T cells. The expression of T-bet was decreased while the expression of Eomes, a marker of exhausted terminal progeny T cells [22], was upregulated in PD1^Hi^ CD8^+^ T cells (Fig. 2a and c). In addition, PD1^Hi^ CD8^+^ T cells also highly expressed BATF, IRF4, NFATC1 and c-MAF (Fig. 2a and c**,** Additional file 4: Figure S3A), which are all implicated in exhausted T cell differentiation [10, 21]. Third, the cytotoxic molecules, including Granzyme B, Granzyme K, Perforin and Granulysin, were strongly decreased in PD1^Hi^ CD8^+^ T cells (Fig. 2a and d**)**, suggesting a compromised killing capacity of these TILs. Taken together, the expression profile of inhibitory receptors, transcription factors and functional molecules ascribed PD1^Hi^CD8^+^ TILs as exhausted T cells.

We further explored the phenotypic characteristics of PD1^Hi^ CD8^+^ T cells and uncovered several key features (Fig. 2e and f, Additional file 4: Figure S3 and Additional file 5: Figure S4). First, PD1^Hi^ CD8^+^ T cells expressed high levels of CD45RO and CD95 and low levels of CD45RA, CCR7, CD57 and KLRG1 suggesting that PD1^Hi^ CD8^+^ T cells are linked with memory T cells but not associated with senescence. Second, certain activation/co-stimulatory markers including HLADR, ICOS, CD28, CD38, CD54, CD69, CD71, CD84, CD98 and 4-1BB were highly expressed, while the expression of other co-stimulatory markers CD6, CD26 and CD44 was down-regulated in PD1^Hi^ CD8^+^ T cells, indicating an unbalanced activation status of these T cells. Third, PD1^Hi^ CD8^+^ T cells highly expressed a panel of markers of cell adhesion and tissue positioning, including CD69 and integrins CD11c, CD49a, CD49b and CD103. Interestingly, most of these markers are pivotal to identify tissue resident memory T cells (Trms), thus PD1^Hi^ CD8^+^ T cells are closely related to Trms. In addition, PD1^Hi^ CD8^+^ T cells preferentially expressed CCR8, CCR10, CXCR3 and CXCR6, but down-regulated the expression of CCR2, CCR5 and CXCR4, suggesting that they have a unique trafficking potential. Finally, PD1^Hi^ CD8^+^ T cells had decreased expression of anti-apoptotic molecule BCL2 and increased expression of pro-apoptotic molecule BAX, as well as increased expression of proliferative marker Ki-67. PD1^Hi^ CD8^+^ T cells also downregulated the expression of transcription factors c-Myc, HIF-1 and HELIOS. c-Myc and HIF-1 are the two TFs important for metabolic reprogramming [23, 24]. HELIOS is associated with T cell activation and cellular division [25]. So, PD1^Hi^ CD8^+^ T cells seem to be less metabolically active and prone to apoptosis. Collectively, a comprehensive characterization of PD1^Hi^ CD8^+^ T cells indicates that these T cells represent exhausted T cells which share features with tissue resident memory T cells and are characterized in an aberrantly activated status with apoptosis-prone potential.

Recent studies have defined exhausted T cells at different stages: progenitor exhausted T cells were TCF1^+^TIM3^−^ with intermediate PD1 expression, while terminal exhausted T cells were TCF1^−^TIM3^+^ and expressed high levels of PD1 [22, 26]. Here we found that PD1^+^ CD8^+^ T cells can be further divided into 3 subsets: PD1^Int^, TIM3^−^PD1^Hi^ and TIM3^+^PD1^Hi^ CD8^+^ T cells (Additional file 5: Figure S4C). The PD1^Int^ CD8^+^ T cells shared the features as progenitor exhausted T cells and TIM3^+^PD1^Hi^ CD8^+^ T cells as terminally exhausted CD8^+^ T cells (Additional file 5: Figure S4C-F). Interestingly, we observed that TIM3^−^PD1^Hi^ CD8^+^ T cells represented a distinct subpopulation of exhausted CD8^+^ T cells that expressed moderate levels of inhibitory receptor (CTLA4, TIGIT, LAG3, CD244 and CD39), exhausted related transcription factors (Eomes, T-bet, Blimp1 and TCF1), activated marker (ICOS, HLADR and 4-1BB) and CD107a expression when compared to PD1^Int^ (progenitor exhausted) and TIM3^+^PD1^Hi^ (terminal exhausted) CD8^+^ exhausted T cells (Additional file 5: Figure S4C-G). Collectively, these results indicate that TIM3^−^PD1^Hi^ CD8^+^ exhausted T cells are most likely at a transitional status (transitional exhausted T cells) differentiated from PD1^Int^ to TIM3^+^PD1^Hi^ CD8^+^ terminally exhausted T cells.

### Decreased pro-inflammatory cytokine-producing capacity of PD1^Hi^ CD8^+^ T cells in HCC

**Fig. 3:**
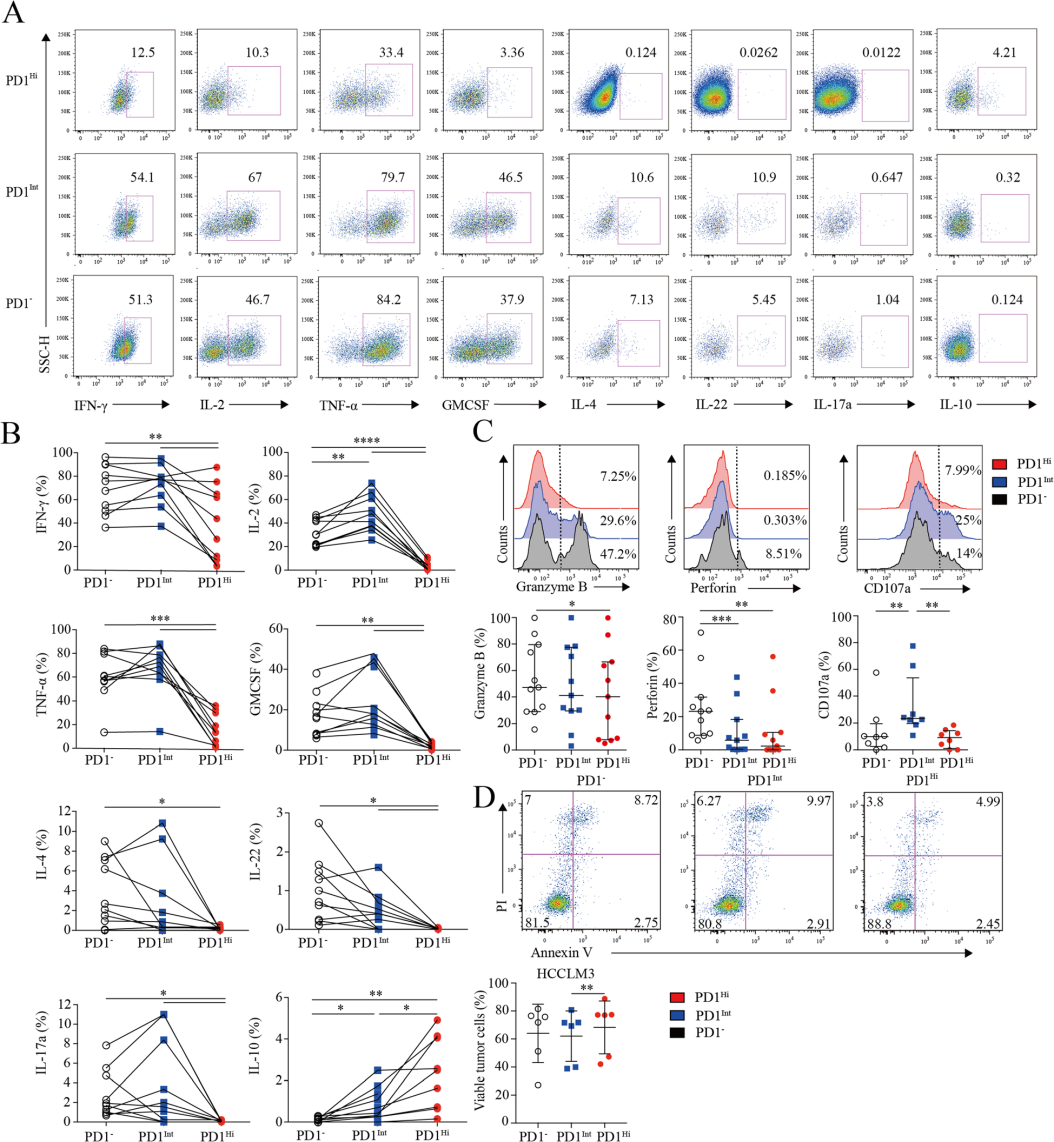
Assessment of pro/anti-inflammatory cytokines profiling and cytotoxic potential of tumor infiltrating PD1^Hi^ CD8^+^ T cells. **a-b**, Representative flow cytometric plots (**a**) and accumulated data (**b**) to show the pro-inflammatory cytokines IFN-γ, IL-2, TNF-α, GM-CSF, IL-4, IL-22, IL-17a and anti-inflammatory IL-10 secreting profile of tumor infiltrating PD1^Hi^, PD1^Int^ and PD1^−^ CD8^+^ T cells following the stimulation of PMA, ionomycin and BFA for 5 h (*n* = 9). **c**, Representative flow cytometric overlays of intracellular Granzyme B, perforin and CD107a expression of tumor infiltrating PD1^Hi^, PD1^Int^, and PD1^−^ CD8^+^ T cells. Granzyme B and perforin were detected from fresh samples (*n* = 11) and CD107a expression was measured after overnight stimulation of coated anti-CD3 (10 μg/mL) and soluble anti-CD28 mAb (1 μg/mL) (*n* = 8). **d**, Apoptosis quantification of HCCLM3 tumor cell line after co-culture with anti-CD3 (10 μg/mL)/CD28 mAb (1 μg/mL) stimulated CD8^+^ T cells subsets for 18 h and pooled statistics of viable HCCLM3 tumor cells shown in bar graph (*n* = 6). Significance was assessed by Wilcoxon matched-pairs signed rank test. *, *P* < 0.05; **, *P* < 0.01; ***, *P* < 0.001; and ****, *P* < 0.0001

Next, we investigated the cytokine-producing capacity of CD8^+^ TILs based on PD1 expression. CD8^+^ TILs from 9 HCC patients were stimulated by PMA and ionomycin in the presence of Brefeldin A, followed by cytokine measurement. As is known, IL-2 is the first compromised cytokine after T cell exhaustion [27]. We observed that the frequency of IL-2-producing PD1^Hi^ CD8^+^ T cells (median = 2.89, 1.11–5.88%) was 10–15 times fewer than those of PD1^Int^ (median = 44.56, 36.54–62.20%; *P* <  0.0001) and PD1^−^ CD8^+^ T cells (median = 30.21, 21.24–43.27%; *P* < 0.0001) (Fig. 3a and b). Furthermore, PD1^Hi^ CD8^+^ T cells exhibited defective production of IFN-γ and TNF-α (median = 35.09, 7.44–67.41%; median = 13.47, 2.12–30.58%), which are typical Th1 cytokines essential for effective anti-tumor responses, in comparison with the PD1^Int^ (median = 77.01, 61.25–82.34%; *P* = 0.0059; median = 70.67, 60.70–80.60%; *P* = 0.002) and PD1^−^ CD8^+^ T cell (median = 71.74, 49.70–90.09%; *P* = 0.002; median = 58.74, 54.47–80.20%; *P* = 0.002). Meanwhile, we also found that PD1^Hi^ CD8^+^ TILs produced much less IL-4, IL-17A, IL-22 and GM-CSF than PD1^Int^ and PD1^−^ CD8^+^ TILs (Fig. 3a and b). IL-10 is a potent immune-suppressive cytokine and contributes to the induction of B7-H1 (PDL1) on monocytes [12, 13]. Notably, the frequency of IL-10-producing cells was elevated to 2.57% (0.69–4.52%) in PD1^Hi^ CD8^+^ T cells, which was significantly higher than PD1^Int^ (median = 0.7, 0.26–1.55%; *P* = 0.002) and PD1^−^ CD8^+^ TILs (median = 0.1, 0–0.21%; *P* = 0.002) (Fig. 3a and b).

Furthermore, PD1^Hi^ CD8^+^ T cells displayed a compromised killing capacity, including decreased expression of lytic effector molecules Granzyme B and perforin (Fig. 3c). Moreover, PD1^Hi^ CD8^+^ T cells expressed considerably lower surface cytotoxic degranulation marker CD107a than PD1^Int^ CD8^+^ T cells upon anti-CD3/CD28 stimulations (Fig. 3c). Thus, under the same anti-CD3 and anti-CD28 stimulation system, three subpopulations of CD8^+^ T cells were sorted by Aria II (BD) with purity > 90% (Additional file 7: Figure S5) and co-cultured in U-bottom plate with HCCLM3 HCC tumor cell line pre-labeled with CFSE according to the manufacture’s instruction. After 18 h of co-culture, cells were stained with Annexin V and PI to evaluate the killing ability of PD1^Hi^CD8^+^ T cells. We prove that HCCLM3 tumor cell line co-cultured with PD1^Int^CD8^+^ T cells were significantly vulnerable to apoptosis than co-cultured with PD1^Hi^CD8^+^ T cells (Fig. 3d**)**. Altogether, these results suggest that PD-1^Hi^ CD8^+^ T cell population was poorly effective in killing cancer cells. Thus, the high expression of PD1 on CD8^+^ TILs is associated with the compromised ability to produce pro-inflammatory cytokines and inferior anti-tumor activity but with the increased capacity to secrete immunosuppressive cytokine IL-10.

### Characterization of PD-1 and TIM-3 expression on CD8^+^ TILs via in situ multispectral imaging

**Fig. 4:**
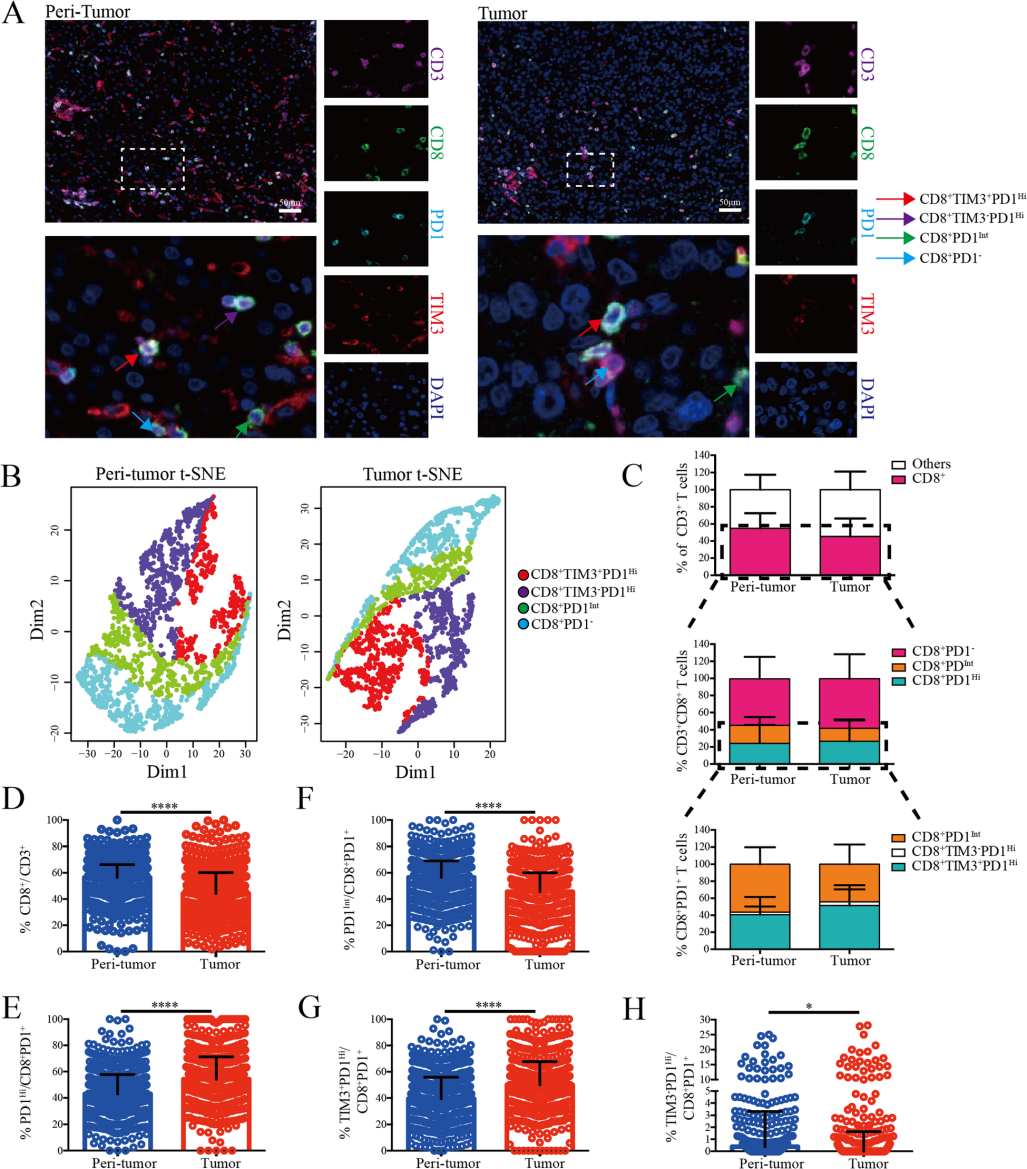
Increased infiltration of exhausted PD1^Hi^ CD8^+^ T cells in HCC tumor tissues revealed by multiplex immunohistochemistry. **a**, Representative multiplex immunofluorescence images to show the distribution of CD8^+^ T cell subsets in peri-tumor and tumor: red arrows (CD8^+^TIM3^+^PD1^Hi^), purple arrow (CD8^+^TIM3^−^PD1^Hi^), green arrow (CD8^+^PD1^Int^) and cyan arrow (CD8^+^PD1^−^). Tissue slides were stained by TSA method and scanned at 20x by Vectra3.0 Automated Imaging System. Scale bar, 50 μm. **b**, t-SNE analysis of CD8^+^ T cells from paired peri-tumor and tumor tissues validated the distinct classification of four CD8^+^ T cell subsets. **c**, Relative distribution analysis of total CD3^+^ T cells in paired peri-tumor and tumor tissues by dividing the total CD3^+^ T cells into CD8^+^ (cytotoxic T cells) and other cells (upper panel). CD8^+^ T cells were further split into CD8^+^PD1^−^, CD8^+^PD1^Int^ and CD8^+^PD1^Hi^ subpopulations (middle panel); and finally CD8^+^PD1^+^ T cells were separated into CD8^+^TIM3^+^PD1^Hi^ and CD8^+^TIM3^−^PD1^Int^ basing on the TIM3 expression (lower panel). **d-h,** Comparisons of the proportions of CD8^+^ among CD3^+^ T cells (**d)** and CD8^+^PD1^Hi^ (**e)**, CD8^+^PD1^Int^ (**f)**, CD8^+^TIM3^+^PD1^Hi^ (**g)** and CD8^+^TIM3^−^PD1^Hi^ (**h)** among CD8^+^PD1^+^ T cells between paired peri-tumor and tumor tissues in training cohort (*n* = 358). Error bars indicated median with interquartile range. Significance was assessed by Wilcoxon matched-pairs signed rank test. *, *P* < 0.05; **, *P* < 0.01; ***, *P* < 0.001; and ****, *P* < 0.0001

Subsequently, we applied multispectral staining to depict CD8^+^ TILs within tumor and peri-tumor of HCC patients (Additional file 8: Figure S6A). Analogous to the flow cytometry, we could classify CD8^+^ TILs into 3 distinct subsets as PD1^Hi^, PD1^Int^, and PD1^−^ based on the PD1 expression. Furthermore, PD1^Hi^ CD8^+^ TILs can be subdivided into TIM3^+^PD1^Hi^ and TIM3^−^PD1^Hi^ when TIM3 was added (Fig. 4a). As expected, we demonstrated the distinct classification of these four CD8^+^ T cells subpopulation by t-SNE (Fig. 4b). Then, we applied this panel on TMA comprising tumor and peri-tumor from two independent cohorts of 358 and 254 HCC patients as training and validation sets, respectively (Additional file 8: Figure S6B). PD1 ‘high’ and TIM3 ‘positive’ thresholds were identified with mean pixel intensity plotting, gated on CD3^+^CD8^+^ T cells (Additional file 8: Figure S6C). The clinicopathologic characteristics of two cohorts were detailed in Additional file 9: Table S2. No significant differences in clinicopathologic features were observed between the two cohorts. The 1, 3, 5-year Overall Survival (OS) rates were 93%, 64%, 40% and 83%, 59%, 51% for the training and validation cohorts, respectively.

When we look into the whole tumor and peri-tumor cores, the proportion of CD8^+^ within CD3^+^ (median = 56.45, 44.33–66.30%) was significantly abundant in peri-tumor compared to tumor (median = 44.26, 29.22–60.16%; *P* < 0.0001) (Fig. 4c and d). In addition, the proportion of PD1^Hi^ CD8^+^ T cells within CD8^+^PD1^+^ T cells (median = 54.23, 39.99–71.33%) was significantly higher in tumor than that of peri-tumor (median = 43.08, 30.33–57.76%; *P* < 0.0001) (Fig. 4e), while the proportions of PD1^Int^ CD8^+^ T cells were opposite (Tumor, median = 45.65, 28.33–60.00%; peri-tumor, median = 56.57, 41.85–69.14%; *P* < 0.0001) (Fig. 4f). In addition, TIM3^+^PD1^Hi^ CD8^+^ T cells represent the majority of PD1^Hi^ CD8^+^ T cells both in tumor and peri-tumor (Fig. 4c). Similar to TIM3^+^PD1^Hi^ CD8^+^ T cells, significantly higher proportions of CD8^+^TIM3^+^PD1^Hi^ within CD8^+^PD1^+^ were detected in tumor (median = 50, 34.57–67.72%) compared to peri-tumor (median = 39.47, 26–55.86%; *P* < 0.0001) (Fig. 4g), while the proportion of CD8^+^TIM3^−^PD1^Hi^ within CD8^+^PD1^+^ was significantly lower in tumor than that in peri-tumor (Fig. 4h). Similar distribution was also observed in the validation cohort (Additional file 8: Figure S6D). Collectively, these results further confirmed the abundant infiltration of CD8^+^PD1^Hi^ and CD8^+^TIM3^+^PD1^Hi^ T cells in HCC.

### Prognostic values of PD1 and TIM3 expression on CD8^+^ TILs in HCC patients

**Fig. 5:**
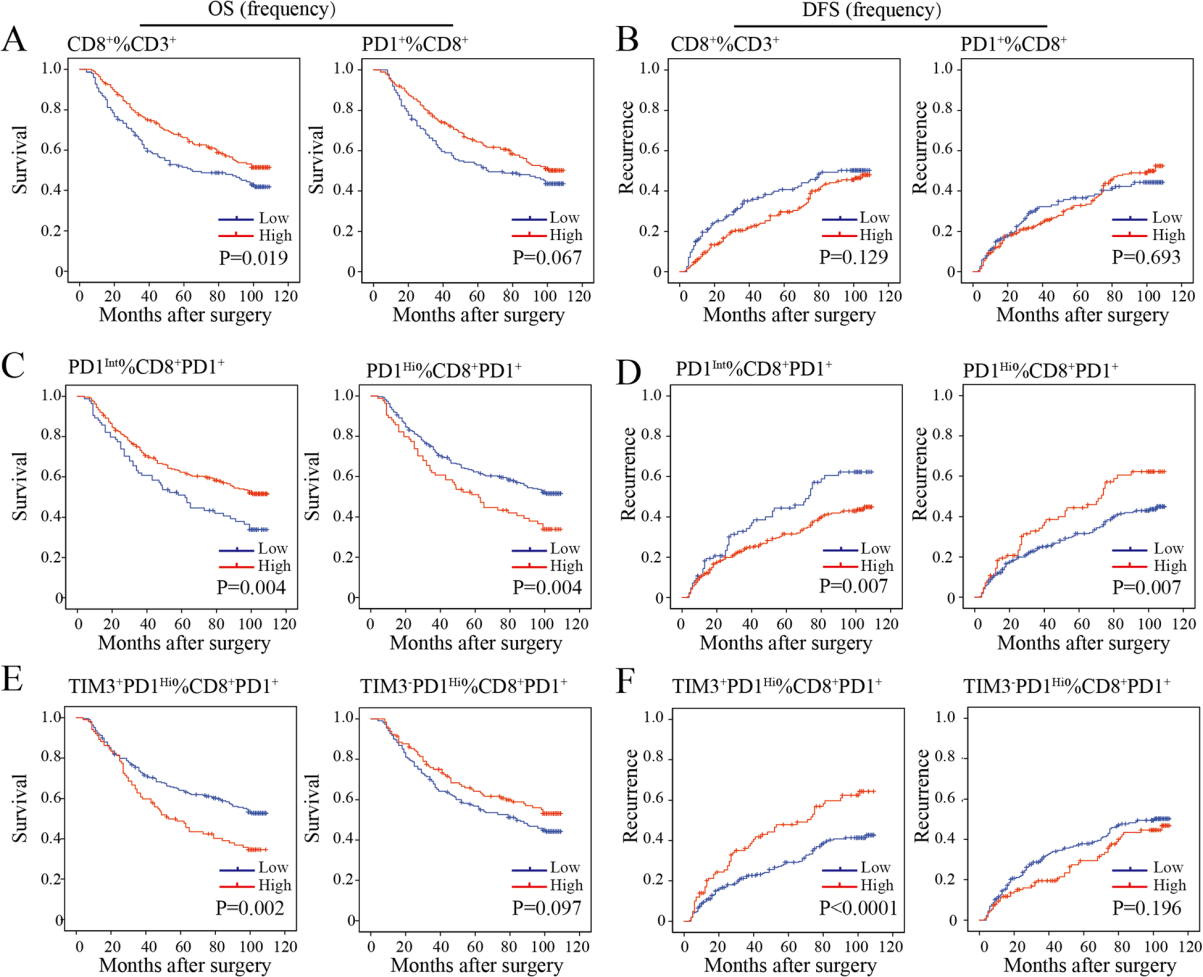
Prognostic significance of the subsets of CD8^+^ TILs in the training cohort. **a-f**, Kaplan-Meier analysis of overall survival (OS) (**a, c, e**) and relapse free survival (RFS) (**b, d, f**) in HCC tumors according to the proportion of CD8^+^ among CD3^+^ TILs and CD8^+^PD1^+^ among CD8^+^ TILs (**a and b**), CD8^+^PD1^Int^ and CD8^+^PD1^Hi^ among CD8^+^PD1^+^ TILs (**c and d**) and CD8^+^TIM3^−^PD1^Hi^ and CD8^+^TIM3^+^PD1^Hi^ among CD8^+^PD1^+^ TILs (**e and f**) in the TMA training cohort (*n* = 358)

We then sought to define whether the infiltration of PD1^Hi^ CD8^+^ T cells in HCC was related to patient survival. We calculated the percentages of defined subpopulations within total CD3^+^ T cells, CD8^+^ T cells, and CD8^+^PD1^+^ T cells successively for each patient. Then, patients were stratified into high and low groups according to the highest Youden index to achieve the optimal cutoffs of each T cell subpopulation. We found that a high proportion of CD8^+^ T cells within CD3^+^ T cells in tumor was associated with prolonged OS (*P* = 0.019) but not relapse-free survival (RFS, *P* = 0.129) (Fig. 5a and b). However, high proportions of PD1^+^ cells among CD8^+^ T cells neither correlated with patients’ OS (*P* = 0.067) nor RFS (*P* = 0.693) (Fig. 5a and b). However, patients with a high proportion of CD8^+^PD1^Hi^ within CD8^+^PD1^+^ correlated with a significantly poor OS (*P* = 0.004) and RFS (*P* = 0.007). Moreover, a higher proportion of CD8^+^TIM3^+^PD1^Hi^ within CD8^+^PD1^+^ showed more striking tendency on OS (*P* = 0.002) and RFS (*P* < 0.0001) than the proportion of CD8^+^PD1^Hi^ within CD8^+^PD1^+^ TILs. In contrast, we observed that a high proportion of CD8^+^PD1^Int^ within CD8^+^PD1^+^ were more likely to have better OS (*P* = 0.004) and RFS (*P* = 0.007) (Fig. 5c-f). Prognostic significance of these TIL subgroups in the validation cohort was similar with that in the training cohort (Additional file 10: Figure S7A-D).

Next, multivariate Cox regression analysis identified that the proportion of PD1^Hi^ (hazard ratio [HR], 1.46; 95% confidence interval [CI], 1.06–2.01; *P* = 0.022), TIM3^+^PD1^Hi^ (HR, 1.48; 95%CI, 1.09–2.01; *P* = 0.013) and PD1^Int^ (HR, 0.69; 95% CI, 0.50–0.95; *P* = 0.022) among CD8^+^PD1^+^ T cells were independent prognostic indices for OS (Table 1). Similarly, the results were observed in the validation cohort (Additional file 11: Table S3). The results indicated that both the proportions of CD8^+^PD1^Hi^ and CD8^+^TIM3^+^PD1^Hi^ within CD8^+^PD1^+^ T cells were independent risk factors for dismal postoperative survival.

**Table 1:** Univariate and Multivariate analysis in the training cohort (*n* = 358)

Variables	Univariate analysis	Multivariate analysis					
		A		B		C	
	*P*	HR (95%CI)	*P*	HR (95%CI)	*P*	HR (95%CI)	*P*
Liver cirrhosis (no vs. yes)	**0.030**	0.816 (0.418–1.591)	0.550	0.816 (0.418–1.591)	0.550	0.871 (0.449–1.688)	0.682
Serum ALT, U/L (≤ 40 vs. > 40)	**0.001**	0.534 (0.335–0.848)	**0.008**	0.534 (0.335–0.848)	**0.008**	0.552 (0.346–0.879)	**0.012**
γ-GT, U/L (> 75 vs. ≤ 75)	**0.023**	1.295 (0.883–1.898)	0.185	1.295 (0.883–1.898)	0.185	1.257 (0.856–1.846)	0.243
Tumor size (cm) (> 5 vs. ≤ 5)	**< 0.0001**	1.984 (1.461–2.696)	**< 0.0001**	1.984 (1.461–2.696)	**< 0.0001**	2.006 (1.474–2.729)	**< 0.0001**
Tumor number (multiple vs. single)	**0.001**	1.451 (1.006–2.092)	**0.046**	1.451 (1.006–2.092)	**0.046**	1.432 (0.993–2.064)	0.054
Vascular invasion (yes vs. no)	**< 0.0001**	1.701 (1.254–2.307)	**0.001**	1.701 (1.254–2.307)	**0.001**	1.695 (1.250–2.300)	**0.001**
TNM stage (I vs. II-III)	**< 0.0001**	0.799 (0.388–1.647)	0.543	0.799 (0.388–1.647)	0.543	0.831 (0.404–1.710)	0.615
CD8^+^PD1^Int^/ CD8^+^PD1^+^ (high vs. low)	**0.005**	0.686 (0.497–0.947)	**0.022**				
CD8^+^PD1^Hi^/ CD8^+^PD1^+^ (high vs. low)	**0.005**			1.458 (1.056–2.013)	**0.022**		
CD8^+^TIM3^+^ PD1^Hi^/ CD8^+^PD1^+^ (high vs. low)	**0.002**					1.476 (1.085–2.008)	**0.013**

Abbreviations: *ALT* Alanine transaminase, *γ-GT* Gamma-Glutamyl-transpeptidase, *TNM* Tumor-nodes-metastases, *HR* Hazard ratio, *CI* Confidential interval. A:CD8^+^PD1^Int^/ CD8^+^PD1^+^; B:CD8^+^PD1^Hi^/ CD8^+^PD1^+^; C:CD8^+^TIM3^+^PD1^Hi^/ CD8^+^PD1^+^. Multivariate analysis was performed by the Cox multivariate proportional hazard regression model with stepwise manner

### Spatial analysis between CD8^+^ T cell subsets and PD-L1^+^ tumor associated macrophages

**Fig. 6:**
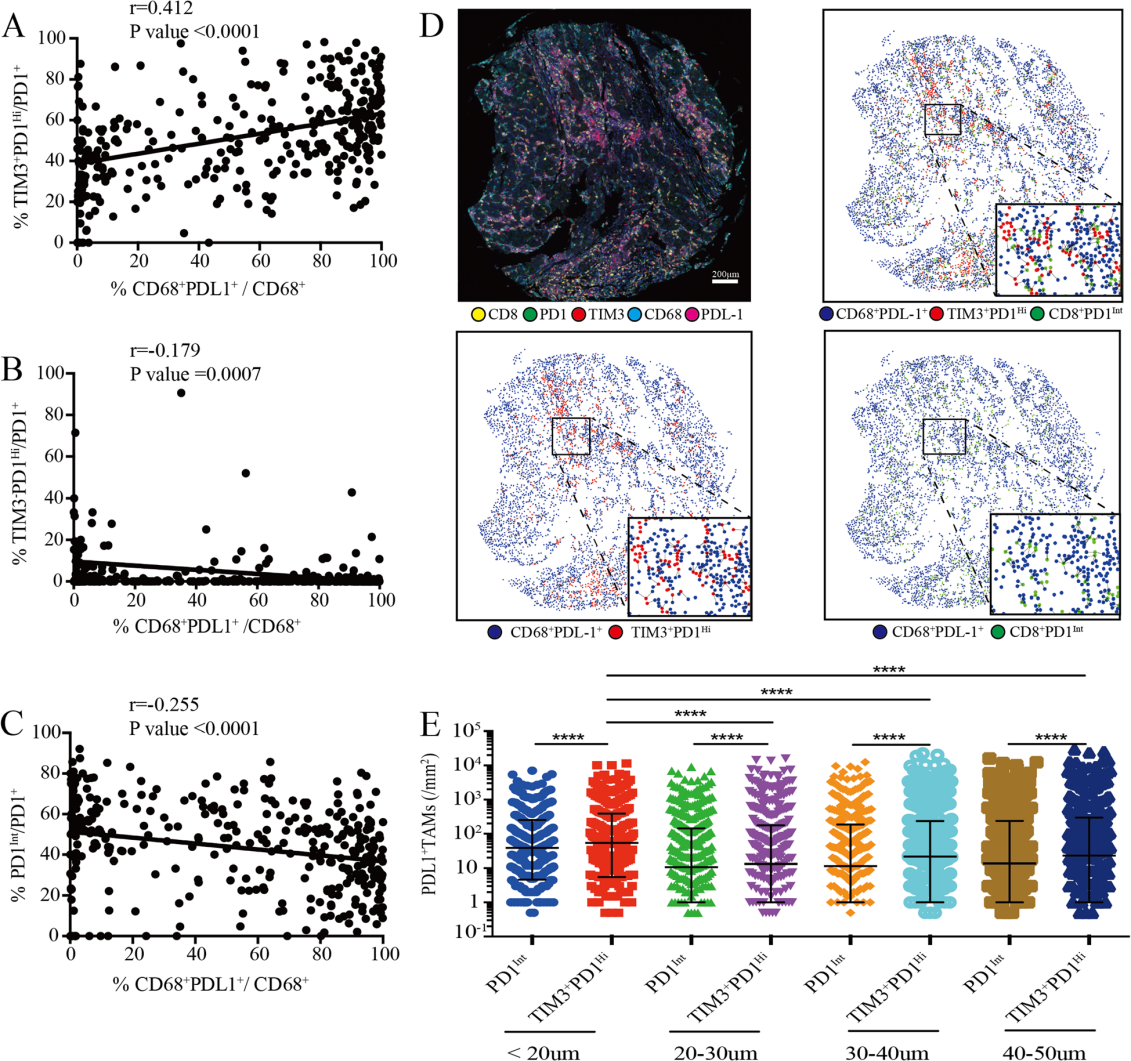
Association of tumor-associated macrophages infiltration with PD-1^Int^ and TIM3^+^PD1^Hi^ CD8^+^ TILs. **a-c**, Correlation analysis between the proportion of tumor infiltrating CD8^+^TIM3^+^PD1^Hi^ (**a**), CD8^+^TIM3^−^PD1^Hi^ (**b**) and CD8^+^PD1^Int^ (**c**) among CD8^+^PD1^+^ TILs and the proportion of PDL1^+^ TAMs within CD68^+^ macrophages per core respectively. Correlation is evaluated by the Spearman correlation coefficient. **d**, Representative multiplex immune-fluorescence image to show the staining for CD8 (yellow), PD1 (green), TIM3 (red), CD68 (cyan), PDL1 (magenta) in the HCC tumor tissue. Cellular phenotype of the fluorescence image depicted the spatial location of CD68^+^PDL1^+^ (blue dots), CD8^+^TIM3^+^PD1^Hi^ (red dots), CD8^+^PD1^Int^ (green dots) in the *situ* tumor tissue. Solid plots and dash line connected the nearest cells within 20 μm from the CD8^+^TIM3^+^PD1^Hi^ and CD8^+^PD1^Int^ to the CD68^+^PDL1^+^ respectively. Scale bar, 200 μm. (**e**) The infiltrating density of PDL1^+^ TAMs within the indicated hierarchy distances of CD8^+^TIM3^+^PD1^Hi^ and CD8^+^PD1^Int^ in the HCC tumor tissues, respectively. Error bars indicated median with interquartile range. Significance was assessed by Wilcoxon matched-pairs signed rank test. ****, *P* < 0.0001. TAMs: tumor associated macrophages

Previously, it has been shown that PD-L1 and Galentin9, the ligands of PD1 and TIM3 respectively, were primarily expressed on tumor cells and CD68^+^ tumor associated macrophages (TAMs) in HCC that promoted immune escape [14, 28]. Strikingly, we found that the proportions of TIM3^+^PD1^Hi^ CD8^+^ TILs were positively correlated with the frequency of PD-L1^+^ TAMs (r = 0.4121; *P* < 0.0001) (Fig. 6a). By contrast, the proportions of both TIM3^−^PD1^Hi^ and PD1^Int^ CD8^+^ TILs showed negative correlations with PD-L1^+^ TAMs (r = − 0.1792; *P* = 0.0007; r = − 0.2551; *P* < 0.0001; respectively) (Fig. 6b and c). Moreover, PD-L1^+^ tumor cells (PDL1^+^CD68^−^) were weakly or not correlated with these three T cell subsets (Additional file 10: Figure S7E). We thus speculated that PD-L1^+^ TAMs, but not PDL1^+^ tumor cells, were positioned near to specific CD8^+^ T cell subsets in order to exert an inhibitory effect. Then we performed spatial analysis and calculated the relative number of PD-L1^+^ TAMs from each PD1^Int^ and TIM3^+^PD1^Hi^ CD8^+^ TIL within a series of distances from 20 to 50 μm (Fig. 6d). We found that in all distances studied, significantly higher numbers of PD-L1^+^ TAMs were around TIM3^+^PD1^Hi^ than those around PD1^Int^ CD8^+^ TILs (Fig. 6e). Moreover, the densities of PD-L1^+^ TAMs were significantly higher within 20 μm of TIM3^+^PD1^Hi^ TILs in comparison with those above 20 μm (Fig. 6e). Together, our results suggested that PD-L1^+^ TAMs may intimately interact with TIM3^+^PD1^Hi^ CD8^+^ TILs in situ which could jointly dampen the effective anti-tumor immune responses.

## Discussion

Tumor-infiltrating cytotoxic CD8^+^ T cells can specifically suppress tumor growth but often turn to a state of “exhaustion” or “dysfunction”. It remains largely undefined that how CD8^+^ T cell exhaustion contributes to the failed immune control during the development of HCC. In the current study, we found that HCC patients had an increased frequency of tumor-infiltrating CD8^+^ T cells expressing a high level of PD1. Even though a recent study also reported PD1^Hi^ exhausted CD8^+^ T cells in HCC [17], our study uncovered novel features of PD1^Hi^ exhausted CD8^+^ T cells by using different experimental strategies. We demonstrated that these exhausted CD8^+^ T cells were in an aberrantly differentiated status, uniquely positioned and revealed as a useful biomarker to predicting unfavorable outcomes in two independent cohorts of HCC patients.

Exhausted CD8^+^ T cells are characterized as impaired cytotoxicity, decreased pro-inflammatory cytokine production and overexpression of multiple inhibitory receptors accompanied by transcriptional and epigenetic changes [10, 21]. Using a flow cytometry-based protein marker profiling the current study not only confirmed the known exhausted features of this specialized CD8^+^ T cell population, but also revealed novel characteristics. A comprehensive cytokine detection revealed that PD1^Hi^ CD8^+^ T cells not only down-regulate canonical CD8^+^ T cell effector cytokines IFN-γ, IL-2 TNF-α, cytotoxic degranulation marker CD107a and the capacity to kill HCC tumor cell HCCLM3, but also the expression of IL-4, IL-17A and IL-22, suggesting a general defect in cytokine production and anti-tumor ability. However, PD1^Hi^ CD8^+^ T cells up-regulated the expression of the immunosuppressive cytokine IL-10, hinting that PD1^Hi^ CD8^+^ T cells may acquire the ability to directly dampen the immune response. Furthermore, we identified that PD1^Hi^ CD8^+^ T cells were in a paradoxically activated status. While panels of activation/co-stimulatory markers were upregulated on PD1^Hi^ CD8^+^ T cells such as ICOS, HLADR, and 4-1BB, they specifically down-regulated co-stimulatory molecules CD6 and CD26. CD6 plays an essential role in transmitting TCR signaling in a Lat-independent manner and is important for continuation of T cell activation [29]. CD26 delivers potent co-stimulatory T-cell activation signals via binding to caveolin-1 [30] or adenosine deaminase [31] on antigen presenting cells. A recent study reported that CD26^Hi^CD4^+^ T cells exhibit superior anti-tumor activity to CD26^int/−^ CD4^+^ T cells [32]. The reasons for the downregulation of the two markers on PD1^Hi^ CD8^+^ T cells are currently not clear which needs further investigation. PD1^Hi^ CD8^+^ T cells also displayed aberrant features including non-proliferative, apoptosis-prone and metabolically less active. Altogether, PD1^Hi^ CD8^+^ T cells seem to be in a frustrated differentiation status.

The enrichment and retention of PD1^Hi^ CD8^+^ T cells within the tumor tissue raise the question of how these cells are recruited and positioned. We found that PD1^Hi^ CD8^+^ T cells expressed high levels of chemokine receptors CCR8, CCR10, CXCR3, and CXCR6. We and others have reported that tumor tissue expressed ligands for these chemokine receptors [33–36], and the interactions of CXCL10-CXCR3, CCL1-CCR8, CCL28-CCR10, and CXCL16-CXCR6 could play an important role in recruiting CD8^+^ T cells into the tumor tissue. In addition, PD1^Hi^ CD8^+^ T cells expressed CD69, CD103 and CD49a, are the hallmarks of Trms [37] as well as integrins likely, CD11c and CD49b, suggesting a tissue resident feature of exhausted T cells in HCC. Trms are generally associated with enhanced cytotoxicity and effector functions and play an active role in anti-tumoral immunity and cancer immunosurveillance [37]. Interestingly, Trms from lung cancer [38] or breast cancer [39] expressed exhausted T cell markers, thus it is important to further elucidate the relationship between the Trms and exhausted T cells in different tumors.

Notably, the spatial analysis revealed that TIM3^+^PD1^Hi^ CD8^+^ T cells and PD-L1^+^ TAMs were in close proximity, suggesting that the two cell populations may be interactive in vivo. We considered that there could be several meanings to this phenomenon. First, PD-L1^+^ TAMs may play an active role in recruiting CD8^+^ T cells to the tumor tissue by producing chemokines or other inflammatory mediators. Supporting this notion, the density of PD-L1^+^ TAMs was reported to be positively correlated with CD8^+^ T cell infiltration in the HCC microenvironment [28]. Second, PD-L1^+^ TAMs may actively induce the exhaustion of CD8^+^ T cells. The development of exhaustion needs at least two types of signals: the intrinsic signal is from chronic TCR stimulation and the extrinsic signals could be cytokines like IL-6, IL-10 and TGF-β [21]. PD-L1^+^ TAMs could provide both signals by presenting antigens and secreting cytokines. Collectively, the intimate spatial relationship between CD8^+^ exhausted T cells and PD-L1^+^ TAMs suggests that they may form a vicious circle to impede the generation of effective anti-tumor immunity.

The frequency of PD1^Hi^ exhausted CD8^+^ T cells was increased in parallel with tumor stages, suggesting that the severity of exhaustion of CD8^+^ T cells was related to the HCC progression. Furthermore, a key finding of our study was that HCC patients with high proportions of TIM3^+^PD1^Hi^ CD8^+^ TILs showed significantly dismal postoperative survival and high risk of recurrence. Although TIM3^−^PD1^Hi^ CD8^+^ TILs were not significantly correlated with postoperative survival, they might be in the transitional exhaustion stage and could play a vital role in contributing to T cell exhaustion. Similarly, other studies also found that PD1^+^ exhausted CD8^+^ T cells were associated with advanced TNM stages, and poor survival in renal cell carcinoma [40], breast cancer [41], follicular lymphoma [42], and head and neck squamous cell carcinoma [43]. The above studies told two facts: exhausted CD8^+^ T cells could act as a biomarker to identify the most care-demanding patients with poor response to conventional therapies and novel strategies are urgently needed to target exhausted CD8^+^ T cells. Immune-checkpoint blockade (ICB) therapy with a purpose of reversing T cell dysfunction and exhaustion has attracted great attention in recent years. However, its clinical success is unfortunately limited to a minority of patients with cancer [44]. For instance only 15–20% of HCC patients responded to PD1 blockade [6]. What’s more, T cell rejuvenation by ICB may be transient and as shown in a recent clinical trial, about one-third of melanoma patients who initially responded to PD-1 blockade experienced tumor relapse [45]. One of the underlying reasons could be anti-PD1 treatment alone only rescue the less exhausted T cells [46], suggesting that blocking of a single immune checkpoint may be ineffective in practice. As exhausted T cells express multiple inhibitory receptors, one important direction is to use combination strategies to simultaneously block several inhibitory receptors including PD1, CTLA-4, LAG-3, Tim-3 or TIGIT [47]. Exhausted T cells also highly express costimulatory receptors, like ICOS, CD28, 4-1BB shown in this study, so ICB plus co-stimulation agonists targeting these costimulatory receptors are also actively explored in tumor immunotherapy [47]. Furthermore, a recent advance in exhausted T cell study has revealed an epigenetic change causes a cell intrinsic barrier for their rejuvenation [48]. For instance, de novo DNA methylation is accompanied with T cell exhaustion and a DNA-demethylating agent enhances the T cell rejuvenation mediated PD1 blockade and tumor control [49]. Finally, targeting tumor-associated macrophages and regulatory T cells to break the immunosuppressive environment [50] represent an additional means to improve the ICB therapy.

## Conclusions

Our findings suggest that increased expression of PD1 and TIM3 leads to CD8^+^ T cell dysfunction and poor survival of the HCC patients, indicating a necessity to identify these HCC patients for additional therapeutic opportunities. This study also showed that PDL1^+^TAMs, but not tumor cells, may actively interact with exhausted CD8^+^ T cells and induced their dysfunction. Further investigations on the mechanisms of T cell exhaustion should deepen our understanding of the immune-compromised status in HCC patients and provide clues for innovative interventions.

## Supplementary information

**Additional file 1.** Supplemental materials and methods (Additional file 12).

**Additional file 2.** Table1. Anti-human antibodies used in flow cytometry (FACS) and multi-spectral immunohistochemistry.

**Additional file 3.** Figure S1. PD1 expression on HCC infiltrating CD8+ T cells and its clinical associations.

**Additional file 4.** Figure S3. Expression pattern of transcription factors, apoptotic and proliferative markers of PD1^Hi^ CD8+ TILs.

**Additional file 5.** Figure S4. Expression pattern of chemokine receptors of PD1Hi CD8+ TILs and phenotypic characteristics of TIM3-PD1^Hi^ and TIM3+PD1^Hi^ TILs.

**Additional file 6.** Figure S2. Detection of the mRNA expression levels of exhaustion related markers in PD1^Hi^ CD8+TILs.

**Additional file 7.** Figure S5. Sorting strategy of PD1^Hi^ CD8+ TILs.

**Additional file 8.** Figure S6. Enriched exhausted PD1^Hi^ CD8+ T cells in HCC tumors.

**Additional file 9.** Table S2. Clinical characteristics of HCC patients.

**Additional file 10.** Figure S7. Prognostic significance of the subsets of CD8+ TILs in the validation cohort.

**Additional file 11.** Table S3. Univariate and multivariate analysis in the validation cohort (*n*=254).

**Additional file 12.** Table S4. qRT-PCR primer sequences.

**Additional file 13.** Supplementary figure legends.

## Data Availability

The datasets used and analyzed during the current study are available from the corresponding author on reasonable request.

## Abbreviations

CTLA4: Cytotoxic T-lymphocyte antigen 4
HCC: Hepatocellular carcinoma
IL: Interleukin
LAG3: Lymphocyte activation gene 3
OS: Overall survival
PD-1: Programmed cell death 1
PD-L1: Programmed cell death-ligand 1
RFS: Relapse free survival
TAMs: Tumor associated macrophages
TIGIT: T cell immunoreceptor with Ig and ITIM domains
TILs: Tumor-infiltrating lymphocytes
TIM-3: T cell immunoglobulin domain and mucin domain-3
TMA: Tissue microarray
TNM: Tumor-nodes-metastases
