# Supplementary material for: PD1Hi CD8+ T cells correlate with exhausted signature and poor clinical outcome in hepatocellular carcinoma
Source: J Immunother Cancer. 2019 Nov 29;7:331. doi: 10.1186/s40425-019-0814-7 (PMC6884778; doi:10.1186/s40425-019-0814-7)
Supplement: Supplementary file 1 — Additional file 1. Supplemental materials and methods (Additional file 12). [file 40425_2019_814_MOESM1_ESM.docx]

**Supplemental materials and methods**

**Multiplex immunohistochemistry**

In brief, 4um FFPE TMAs sections were deparaffinized in xylene and then rehydrated in 100%, 90%, 70% alcohol successively. Antigen unmasking was performed with a preheated epitope retrieval solution (100X citrate buffer, pH=6), endogenous peroxidase was inactivated by incubation in 3% H_2_O_2_ for 20 min. Next, the sections were pre-incubated with 10% normal goat serum and then incubated overnight with primary antibodies: mouse anti-CD3 antibody, mouse anti-CD8 antibody, mouse anti-PD1 antibody, rabbit anti-TIM3 antibody. Detailed information of antibodies was provided in the **Supplementary Table S1**. Next, sections were incubated with the corresponding HRP-conjugated second antibodies (Vector Lab, CA) for 30 min at room temperature. The antigenic binding sites were visualized using the OPAL dye. Opal- 570 (PerkinElmer Inc.), Opal -690 (PerkinElmer Inc.), Opal -520 (PerkinElmer Inc.), Opal -650 (PerkinElmer Inc.) were applied to each antibody.

**Quantitative analysis**

Images were analyzed and quantified by inForm (v2.3, PerkinElmer Inc.) based active machine learning algorithm with a pre visual cutoff followed by single-cell based mean pixel fluorescence intensity to achieve accuracy. Threshold value of each marker was identified and displayed by both FCS Express 6 Plus v6.04.0034 (De Novo Software) with FACS alike density plot (**Supplementary Fig. S6C**), and Inform Score that could adjust the cutoff based on the score map and original staining images to improve the accuracy. In addition, it also need to combine the expression characteristic of different makers, such as TIM-3 could serve as auxiliary marker to further differentiate PD1^Hi^ and PD1^Int^ T cells. Furthermore, enumeration of cell phenotype and distance analysis processed by R script. Cells that were higher than the threshold of both CD3, CD8 and PD1 were considered PD1^+^CD8^+^ T cells. Cells that were higher than the threshold of CD3, CD8 and PD1^Hi^ were considered PD1^Hi^CD8^+^ T cells. Cells that were positive for the CD3, CD8 and PD1, but lower than the threshold of PD1^Hi^ were considered PD1^Int^CD8^+^ T cells. Except for PDL1^+^CD68^+^ double positive cells, the rest PDL1^+^ cells were considered as HCC tumor cell expressed PDL1. Evaluation of the frequency of PD1^Hi^CD8^+^T cells within PD1^+^CD8^+^T cells, was initially performed by obtaining the absolute number of PD1^Hi^CD8^+^ T cells and then normalized that to the number of PD1^+^CD8^+^T cells according to the following formula: PD1^Hi^% PD1^+^CD8^+^T cell= number of PD1^Hi^CD8^+^T cells/ the number of PD1^+^CD8^+^T cells. Similar formula was applied for others indices. For the analysis of t-SNE, we picked out totally 4000 cells from 4 distinct CD8^+^ T cells subsets and reduce dimension of data to two by R script. Unsupervised clustering analysis was performed by the K-means clustering analysis.

**Isolation of mononuclear cells from peripheral blood and tissues**

Briefly, tissues (n =28) were cut into small pieces and digested in RPMI 1640 (Gibco^®^, 1187509) containing 1mg/ml collagenase IV (Gbicol, 17104019), 0.4mg/ml hyaluronidase and 0.04mg/ml DNase I, on a gentle MACS Octo Dissociator with Heaters machine (Miltenyi Biotec) at 37°C for 1 hour. The suspension of cells was filtered through a 48-μm mesh and separated by Ficoll centrifugation. The mononuclear leukocytes were washed and re-suspended in PBS supplemented with fetal bovine serum for FACS analysis.

**CD107a detection assay**

For CD107a staining, mononuclear cells were cultured in medium alone (as unstimulated background) or stimulated with plate-coated anti-CD3 mAb (10μg/ml, clone HIT3a) and soluble anti CD28 (1μg/ml, clone CD28.2) in 37℃for overnight (14-16 hours) in the presence of PE anti CD107a antibody (1:1000 dilution, clone H4A3, Biolegend) and monension (1μg/ml, Biolegend). Dead cells were excluded by Zombie yellow dye staining (Biolegend) prior to staining with antibodies against surface protein of CD3, CD8, PD1 and TIM3. Unstimulated background was substracted to calculated the frequency of CD107a-positive cells.

**PD1^Hi^CD8^+^ T cell sorting**

Tumor-infiltrating CD8^+^ T cells were isolated using magnetic beads (anti-CD8, Miltenyi Biotech, German) by AutoMACS (Miltenyi Biotech). To obtain PD1^Hi^CD8^+^ T cells, isolated purified CD8^+^ T cells were stained with anti-CD8, anti-PD1 and anti-TIM3. CD8^+^ T cells were further differentiated into PD1-high, PD1-intermediate and PD1 negative based on PD1 MFI level and TIM3 expression and sorted by Aria II (BD) with average purity more than 90%.

**PD1^Hi^CD8^+^ T cell co-culture with HCC cell line and apoptosis assays**

After sorting from 6 HCC patients tumor tissue, three subsets of CD8^+^ T cells were stimulated and allocated to 96-well U-bottom plate (2×10^4^), pre-coated with 10μg/ml purified anti-CD3 antibody (clone HIT3a) and soluable anti-CD28 antibody (1μg/ml, clone CD28.2) in RPMI 1640. Before co-culture, HCCLM3 (RRID:CVCL_6832, Liver Cancer Institute, Fudan University) HCC cell line were labeled with 10μM CFSE (Thermofisher, German) according to manufacturer’s instructions. Next, HCC cell line was co-cultured with PD1^Hi^, PD1^Int^ and PD1^-^ CD8^+^ T cells at ratios of 1:1. After 18 hours in 37°C, CFSE labeled cell were harveasted, tumor cell line survival and apoptosis was quantified by using Annexin V-APC and propidium iodide (PI) (Biolegend) according to the manufacturer’s instructions.

**RNA isolation and quantitative reverse transcription PCR (qRT-PCR)**

Quantitative RT-PCR of the multiple genes including *PDCD1*, *HAVCR2*, *CTLA4*, *LAG3* and *ENTPD1* of different subtypes of CD8^+^ T cells (PD1^-^, PD1^Int^, PD1^Hi^) was accomplished using the method as reported before(1). In brief, based on our FACS analysis, single cells suspension was first collected in PBS supplemented with 1% fetal bovine serum by BD FACS Melody flow cytometric cell sorter (BD). Next, the total cellular RNA was extracted using TRIzol reagent (Invitrogen, 15596018) and cDNA was synthesized using PrimeScript™ RT reagent Kit (Takara, RR037A) according to the manufacturer’s instructions. The gene expression levels were measured by PCR amplification with SYBR Premix EX Taq (Tli RNaseH plus, Takara, RR420A) using target primers Primers listed in **Supplementary Table S4.** Detail procedures of RNA isolation and qRT-PCR are presented in supplemental materials and methods. Targeted gene expression levels were normalized to the values of β-actin. The relative gene expression level was calculated with the 2^-ΔΔCt^ method.

**Reference**

1. Wang S, Xia P, Chen Y, Qu Y, Xiong Z, Ye B, Du Y, et al. Regulatory Innate Lymphoid Cells Control Innate Intestinal Inflammation. Cell 2017;171:201-216 e218.
