# Supplementary material for: PD1Hi CD8+ T cells correlate with exhausted signature and poor clinical outcome in hepatocellular carcinoma
Source: J Immunother Cancer. 2019 Nov 29;7:331. doi: 10.1186/s40425-019-0814-7 (PMC6884778; doi:10.1186/s40425-019-0814-7)
Supplement: Supplementary file 2 — Additional file 2. Table1. Anti-human antibodies used in flow cytometry (FACS) and multi-spectral immunohistochemistry. [file 40425_2019_814_MOESM2_ESM.docx]

**Supplementary Table 1.** Anti-human antibodies used in flow cytometry (FACS) and Multi-spectral immunohistochemistry.

| **Ant­ibody** | **Channel** | **Catalogue or clone** | **Company** |
| --- | --- | --- | --- |
| CD2 | APC | RPA-2.10 | eBioscience |
| CD3 | BUV395 | SK7 | BD |
| CD4 | BV510 | Sk3 | BD |
| CD5 | APC | L17F12 | eBioscience |
| CD6 | PE | BL-CD6 | Biolegend |
| CD7 | PE | CD7-6B7 | eBioscience |
| CD8 | A700 | HIT8a | Biolegend |
| CD9 | FITC | HI9a | Biolegend |
| CD11a | FITC | G43-25B | BD |
| CD11b | APC | M1/70 | eBioscience |
| CD11c | APC | Bu15 | Biolegend |
| CD16 | FITC | eBioCB16 | eBioscience |
| CD26 | APC | BA5b | Biolegend |
| CD27 | FITC | O323 | Biolegend |
| CD28 | APC | CD28.2 | eBioscience |
| CD30 | PE | 12-0309-42 | eBioscience |
| CD31 | PE | WM59 | Biolegend |
| CD38 | APC | 555462 | BD |
| CD39 | PE | A1 | Biolegend |
| CD43 | APC | EBIO-84-3C1 | eBioscience |
| CD44 | PE | BJ18 | Biolegend |
| CD45RA | FITC | HI100 | Biolegend |
| CD45RO | APC | 17-0457 | eBioscience |
| CD49a | A647 | TS2/7 | Biolegend |
| CD49b | APC | P1E6-C5 | Biolegend |
| CD54 | PE | HCD54 | Biolegend |
| CD56 | FITC | 5.1H11 | Biolegend |
| CD57 | FITC | TB01 | eBioscience |
| CD58 | PE | 330908 | Biolegend |
| CD62L | FITC | DREG56 | Biolegend |
| CD69 | FITC | FN50 | BD |
| CD71 | APC | M-A712 | BD |
| CD73 | PE | AD2 | Biolegend |
| CD84 | PE | CD84.1.21 | Biolegend |
| CD95 | FITC | DX2 | Biolegend |
| CD98 | PE | UM7F8 | BD |
| CD103 | BV711 | Ber-ACT8 | BD |
| CD107a | PE | H4A3 | Biolegend |
| CD122 | APC | TU27 | Biolegend |
| CD123 | APC | 5B11 | eBioscience |
| CD124 | PE | mlL4R-M1 | BD |
| CD129 | PE | AH9R7 | Biolegend |
| CD134(OX40) | PE | Ber-ACT35 | Biolegend |
| CD137(4-1BB) | FITC | 4B4(4B4-1) | eBioscience |
| CD152(CTLA4) | PE | 14D3 | eBioscience |
| CD160 | APC | BY55 | Biolegend |
| CD161 | APC | HP-3G10 | eBioscience |
| CD95L(CD178) | PE | NOK-1 | eBioscience |
| CD210 | PE | 3F9 | Biolegend |
| CD223(LAG3) | PE | 3DS223H | eBioscience |
| CD226(TIGIT) | PE | 559788 | BD |
| CD244(2B4) | PE | 2-69 | BD |
| CD268(BAFFR) | PE | 11C1 | Biolegend |
| CD272(BTLA) | PE | MIH26 | Biolegend |
| CD274(PDL1) | APC | 29E.2A3 | Biolegend |
| CD279(PD1) | BV421 | EH12.2H7 | Biolegend |
| CD314 | PE | 1D11 | Biolegend |
| TIM3(CD366) | PE-Cy7 | F38-2E2 | Biolegend |
| ICOS | APC | C398.4A | Biolegend |
| HLADR | FITC | L243 | Biolegend |
| Eomes | PE | WD1928 | eBioscience |
| T-bet | A647 | 4B10 | Biolegend |
| BATF | PE | S39-1060 | BD |
| IRF4 | eF660 | 3E4 | eBioscience |
| Blimp1 | PE | 3h2E8 | novusbi |
| BCL2 | A647 | 658707 | Biolegend |
| BCL6 | PE | K112-91 | BD |
| BCL-XL | A647 | H-62 | SANTACRUZ |
| BID | PE | 104682MM10P | SB |
| BAX | PE | 2D2 | SANTACRUZ |
| C-MAF | eF660 | symOF1 | eBioscience |
| Foxo1 | PE | C29H4 | CST |
| MCL1 | FITC | LVUBKM | eBioscience |
| Gata3 | PE | TWAJ | eBioscience |
| Helios | A647 | 22F6 | eBioscience |
| NFATC1 | PE | 7A6 | Biolegend |
| NFATC2 | PE | D4381 | CST |
| Caspase3 | A647 | C92-605 | BD |
| Akt(pT308) | PE | J1-223.371 | BD |
| Akt(pS4733) | PE | D9E | CST |
| PS6 | APC | cupk43k | eBioscience |
| TCF-1 | A647 | 7F11A10 | Biolegend |
| RUNX3 | PE | R3-5G4 | BD |
| Foxp3 | eF660 | 236A/E7 | eBioscience |
| Ki67 | A647 | Ki-67 | Biolegend |
| c-Myc | PE | D84C12 | CST |
| GranzymeA | APC | CB9 | Biolegend |
| GranzymeB | PE | GB11 | eBioscience |
| GranzymeK | FITC | GM26E7 | Biolegend |
| Perforin | APC | 308112 | Biolegend |
| Granulysin | A488 | DH2 | Biolegend |
| CCR1 | A647 | 53504 | BD |
| CCR2 | PE | KO36C2 | Biolegend |
| CCR3 | APC | eBio5E8-G9-B4 | eBioscience |
| CCR4 | PE | L291H4 | Biolegend |
| CCR5 | APC | J418F1 | Biolegend |
| CCR6 | PE | 559562 | BD |
| CCR7 | FITC | G043H7 | Biolegend |
| CCR8 | PE | FAB1428P | R&D |
| CCR9 | APC | 557975 | BD |
| CCR10 | PE | 1B5 | BD |
| CXCR1 | A488 | 8F1 | Biolegend |
| CXCR2 | A488 | 5E8 | Biolegend |
| CXCR3 | PE | G025H7 | Biolegend |
| CXCR4 | APC | 12G5 | Biolegend |
| CXCR5 | A488 | RF8B2 | BD |
| CXCR6 | A647 | 335101 | Biolegend |
| CXCR7 | PE | 10D1-J16 | Biolegend |
| CX3CR1 | FITC | 2A9-1 | Biolegend |
| IL-1R1 | PE | FAB5231P | R&D |
| IL-1R4 | PE | BAA02233 | R&D |
| IL-23R | PE | 218216 | R&D |
| IFN-AR2 | PE | REA124 | Miltenyi |
| IL-2 | APC | MQ1-17H12 | eBioscience |
| IL-4 | PE | MP4-25D2 | Biolegend |
| IL-10 | PE | JES3-9D7 | Biolegend |
| IL-17A | PE-eFluor610 | BL168 | Biolegend |
| IL-22 | PECy7 | 22URTI | eBioscience |
| IFN-γ | PB | 4S.B3 | Biolegend |
| TNF-α | PECy7 | MAb11 | eBioscience |
| GM-CSF | PC594 | BVD2-21C11 | BD |
| CD3 |  | ab16669, SP7 | Abcam |
| CD8 |  | M7103, C8/144B | Dako |
| TIM3 |  | 45208, D5D5R | CST |
| PD1 |  | 367402, NAT105 | Biolegend |
| CD68 |  | MAB-0041, KP1 | Maixin |
| PDL1 |  | GT220429, SP142 | GeneTech_shanghai |

**Abbreviations：**PD-1, programmed cell death 1; PD-L1, programmed cell death-ligand 1; TIM-3, T cell immunoglobulin domain and mucin domain-3; CTLA4, cytotoxic T-lymphocyte antigen 4; LAG3, lymphocyte activation gene 3; TIGIT, T cell immunoreceptor with Ig and ITIM domains; BTLA, B- and T-lymphocyte attenuator; Eomes, Eomesodermin ; IRF4, Interferon regulatory factor 4; Blimp-1, PR domain zinc finger protein 1; NFATC1, Nuclear factor of activated T-cells, cytoplasmic 1; HLA-DR, Human Leukocyte Antigen– DR isotype; ICOS, inducible costimulator; IL, interleukin; IFN-γ, interferon-γ; TNF-α, tumor necrosis.
