# Supplementary figures and images for: PD1Hi CD8+ T cells correlate with exhausted signature and poor clinical outcome in hepatocellular carcinoma
Source: J Immunother Cancer. 2019 Nov 29;7:331. doi: 10.1186/s40425-019-0814-7 (PMC6884778; doi:10.1186/s40425-019-0814-7)

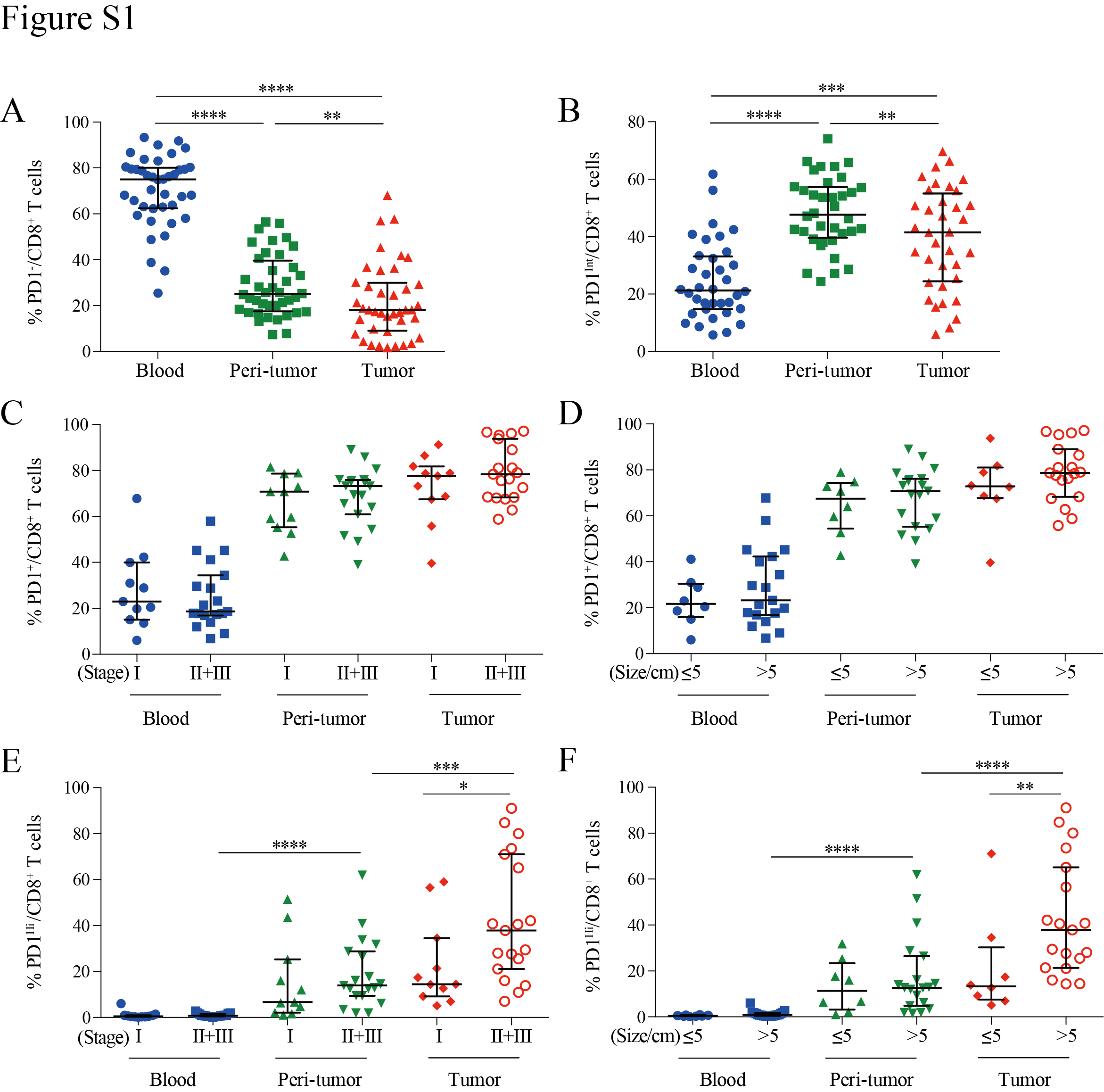

Supplement: Supplementary file 3 — Additional file 3. Figure S1. PD1 expression on HCC infiltrating CD8+ T cells and its clinical associations. [file 40425_2019_814_MOESM3_ESM.tif]

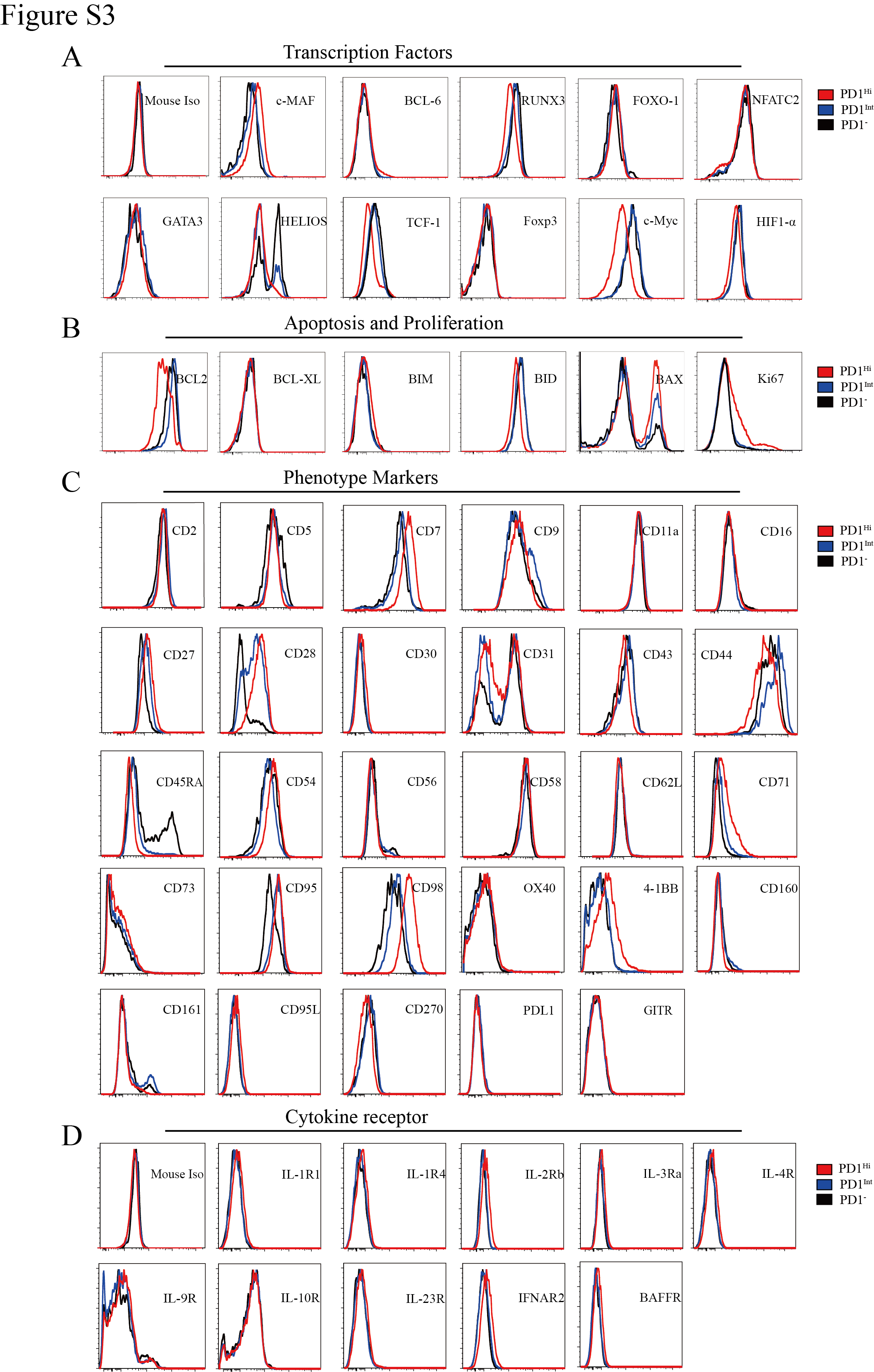

Supplement: Supplementary file 4 — Additional file 4. Figure S3. Expression pattern of transcription factors, apoptotic and proliferative markers of PD1Hi CD8+ TILs. [file 40425_2019_814_MOESM4_ESM.tif]

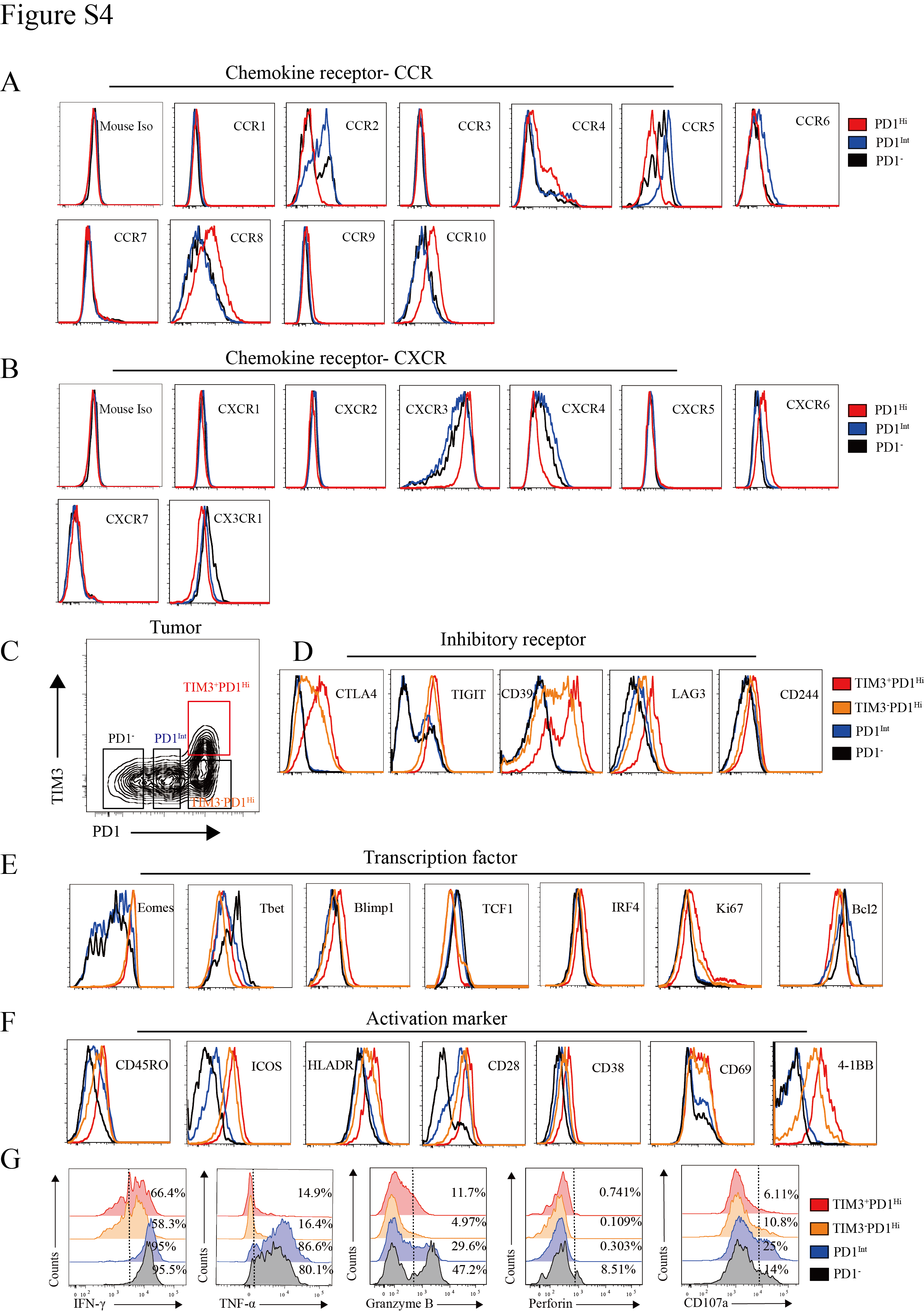

Supplement: Supplementary file 5 — Additional file 5. Figure S4. Expression pattern of chemokine receptors of PD1Hi CD8+ TILs and phenotypic characteristics of TIM3-PD1Hi and TIM3+PD1Hi TILs. [file 40425_2019_814_MOESM5_ESM.tif]

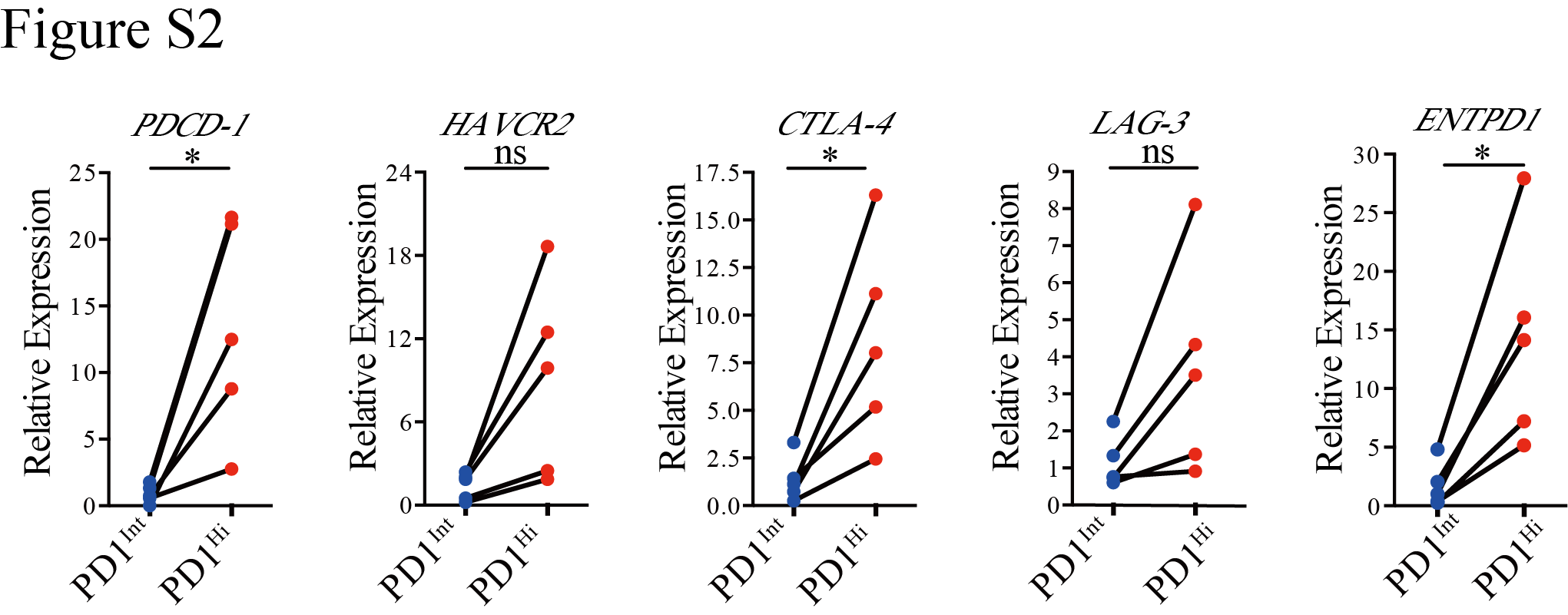

Supplement: Supplementary file 6 — Additional file 6. Figure S2. Detection of the mRNA expression levels of exhaustion related markers in PD1Hi CD8+TILs. [file 40425_2019_814_MOESM6_ESM.tif]

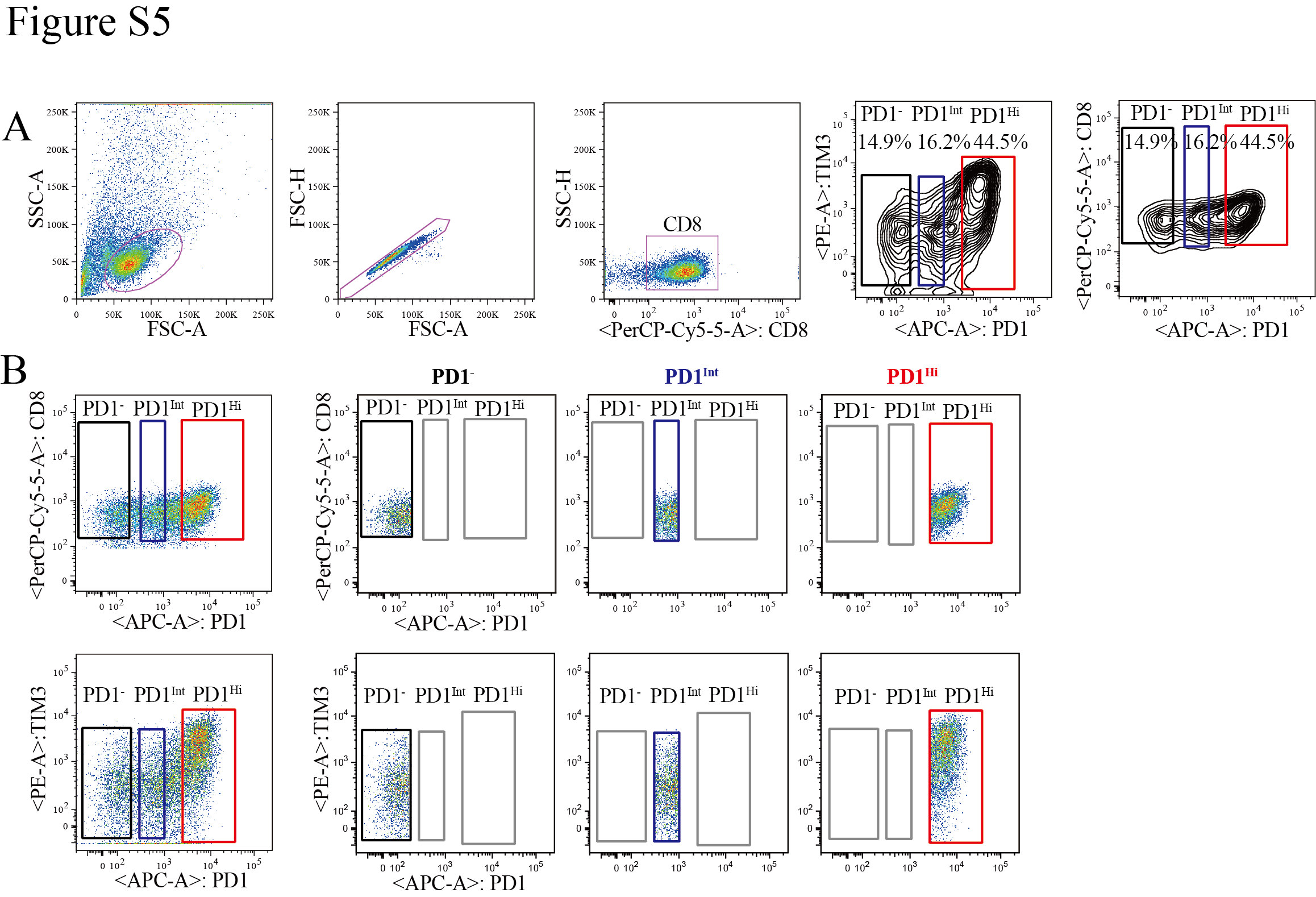

Supplement: Supplementary file 7 — Additional file 7. Figure S5. Sorting strategy of PD1Hi CD8+ TILs. [file 40425_2019_814_MOESM7_ESM.tif]

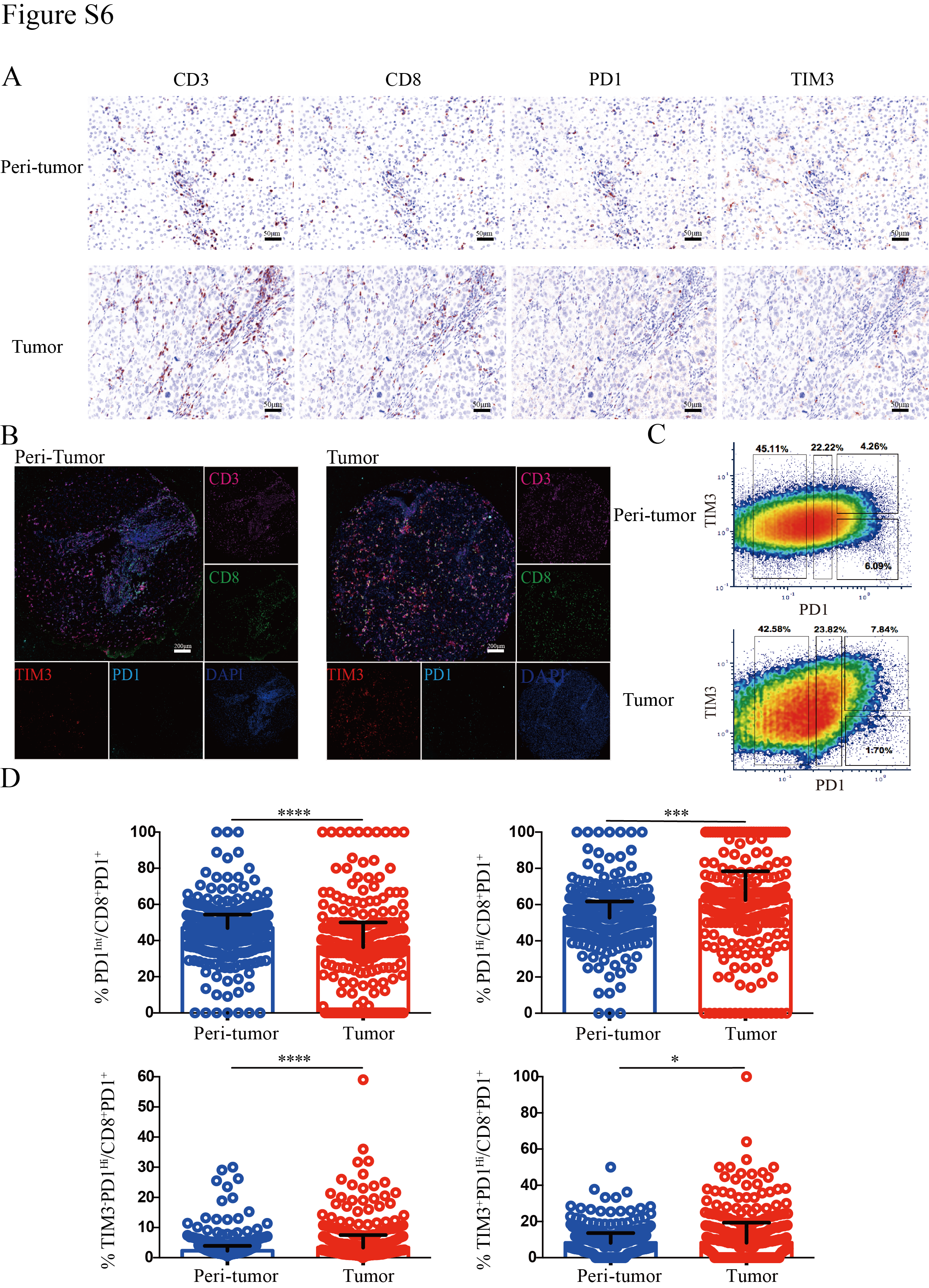

Supplement: Supplementary file 8 — Additional file 8. Figure S6. Enriched exhausted PD1Hi CD8+ T cells in HCC tumors. [file 40425_2019_814_MOESM8_ESM.tif]

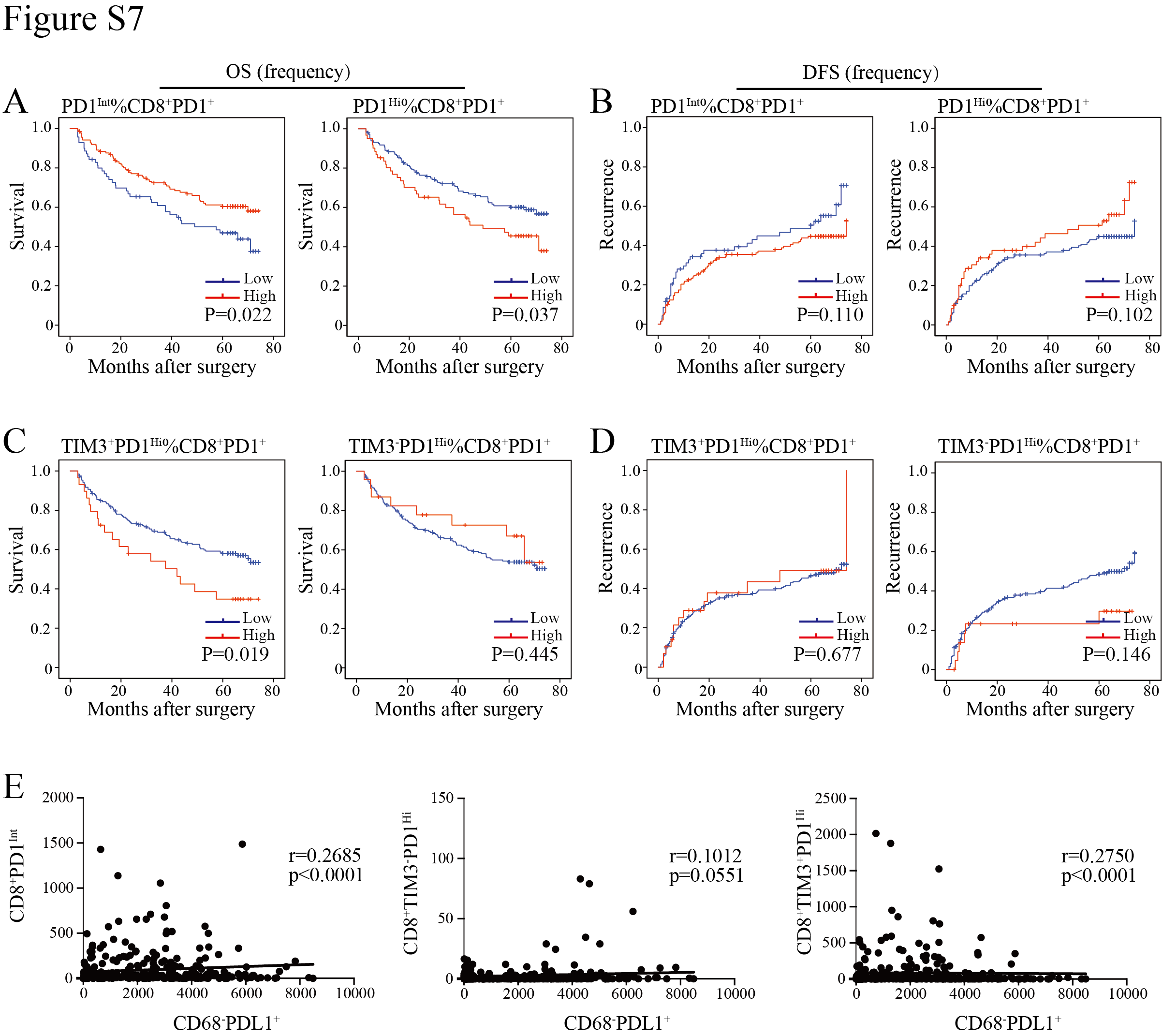

Supplement: Supplementary file 10 — Additional file 10. Figure S7. Prognostic significance of the subsets of CD8+ TILs in the validation cohort. [file 40425_2019_814_MOESM10_ESM.tif]
