# Supplementary material for: PD1Hi CD8+ T cells correlate with exhausted signature and poor clinical outcome in hepatocellular carcinoma
Source: J Immunother Cancer. 2019 Nov 29;7:331. doi: 10.1186/s40425-019-0814-7 (PMC6884778; doi:10.1186/s40425-019-0814-7)
Supplement: Supplementary file 9 — Additional file 9. Table S2. Clinical characteristics of HCC patients. [file 40425_2019_814_MOESM9_ESM.docx]

**Supplementary Table 2.** Clinical characteristics of HCC patients.

| **Variables** | **Training cohort (n=358)** | **Validation cohort (n=254)** | **P value** |
| --- | --- | --- | --- |
| Gender  (male vs. female) | 296/62 | 221/33 | 0.156 |
| HbsAg  (negative vs. positive) | 28/330 | 5/249 | 0.815 |
| Liver cirrhosis  (yes vs. no) | 300/58 | 223/31 | 0.735 |
| Tumor differentiation  (I+II vs. III+IV) | 257/101 | 176/78 | 0.911 |
| Vascular invasion  (yes vs. no) | 117/241 | 91/163 | 0.180 |
| TNM  （I/ II/ III） | 207/116/35 | 138/61/55 | 0.228 |
| BCLC  (0/A/B/C) | 49/110/82/117 | 17/80/65/92 | 0.838 |
| Tumor number  (single vs. multiple) | 302/56 | 208/46 | 0.171 |
| Tumor size (cm)  (≤5 vs. >5) | 226/132 | 116/138 | 0.386 |
| Serum AFP (ng/ml)  (>20 vs. ≤20) | 137/221 | 100/154 | **0.006** |
| Recurrence  (yes vs. no) | 151/207 | 120/134 | 0.332 |
| Death  (yes vs. no) | 183/175 | 116/138 | 0.890 |

**Abbreviations:** HCC, hepatocellular carcinoma; HBsAg, hepatitis B surface antigen; TNM, tumor-nodes-metastases; BCLC, Barcelona Clinic Liver Cancer; AFP, α-fetopr­otein.
