# Supplementary material for: PD1Hi CD8+ T cells correlate with exhausted signature and poor clinical outcome in hepatocellular carcinoma
Source: J Immunother Cancer. 2019 Nov 29;7:331. doi: 10.1186/s40425-019-0814-7 (PMC6884778; doi:10.1186/s40425-019-0814-7)
Supplement: Supplementary file 11 — Additional file 11. Table S3. Univariate and multivariate analysis in the validation cohort (n=254). [file 40425_2019_814_MOESM11_ESM.docx]

**Supplementary Table 3.** Univariate and Multivariate analysis in the validation cohort (n=254)**.**

| **Variables** | **Univariate**  **analysis** | **Multivariate analysis** | | | | | |
| --- | --- | --- | --- | --- | --- | --- | --- |
|  |  | **A** | | **B** | | **C** | |
|  | **P** | **HR**  **(95%CI)** | **P** | **HR**  **(95%CI)** | **P** | **HR**  **(95%CI)** | **P** |
| TB, mol/L  (>22 vs. ≤22) | **0.025** | 2.062  (1.084-3.923) | **0.027** | 2.028  (1.067-3.854) | **0.031** | 1.789  (0.947-3.381) | 0.073 |
| Serum AFP, ng/ml  (>20 vs. ≤20) | **0.001** | 1.294  (0.824-2.032) | 0.263 | 1.331  (0.846-2.095) | 0.216 | 1.276  (1.074-2.418) | 0.275 |
| γ-GT, U/L  (≤ 75 vs. >75) | **0.017** | 0.702  (0.333-1.480) | 0.353 | 0.833  (0.385-1.805) | 0.644 | 0.765  (0.362-1.617) | 0.483 |
| Tumor size (cm)  (> 5 vs. ≤ 5) | **< 0.0001** | 2.286  (1.461-3.577) | **<0.0001** | 2.187  (1.395-3.429) | **0.001** | 2.322  (1.483-3.634) | **<0.0001** |
| Tumor differentiation (III+IV vs. I+II) | **<0.0001** | 1.638  (1.057-2.537) | **0.027** | 1.695  (1.100-2.613) | **0.017** | 1.726  (1.139-2.617) | **0.010** |
| Microvascular invasion  (yes vs. no) | **< 0.0001** | 1.418  (0.650-3.096) | 0.380 | 1.391  (0.639-3.030) | 0.406 | 2.398  (1.557-3.693) | **<0.0001** |
| TNM stage  (III-II vs. I) | **< 0.0001** | 2.382  (1.537-3.689) | **<0.0001** | 2.544  (1.635-3.959) | **<0.0001** | 1.441  (0.719-2.888) | 0.303 |
| CD8^+^PD1^Int^/ CD8^+^PD1^+^  (high vs. low) | **0.024** | 0.550  (0.359-0.842) | **0.006** |  |  |  |  |
| CD8^+^PD1^Hi^/ CD8^+^PD1^+^  (high vs. low) | **0.038** |  |  | 1.665  (1.078-2.573) | **0.022** |  |  |
| CD8^+^TIM3^+^ PD1^Hi^/ CD8^+^PD1^+^  (high vs. low) | **0.021** |  |  |  |  | 2.198  (1.297-3.725) | **0.003** |

**Abbreviations:** TB, total bilirubin; AFP, α-fetoprotein; γ-GT, Gamma-Glutamyl-transpeptidase; TNM, tumor-nodes-metastases; HR, hazard ratio; CI, confidential interval. A:CD8^+^PD1^Int^/ CD8^+^PD1^+^; B: CD8^+^PD1^Hi^/ CD8^+^PD1^+^; C:CD8^+^TIM3^+^PD1^Hi^/ CD8^+^PD1^+^. Multivariate analysis was performed by the Cox multivariate proportional hazard regression model with stepwise manner.
