# Supplementary material for: PD1Hi CD8+ T cells correlate with exhausted signature and poor clinical outcome in hepatocellular carcinoma
Source: J Immunother Cancer. 2019 Nov 29;7:331. doi: 10.1186/s40425-019-0814-7 (PMC6884778; doi:10.1186/s40425-019-0814-7)
Supplement: Supplementary file 12 — Additional file 12. Table S4. qRT-PCR primer sequences. [file 40425_2019_814_MOESM12_ESM.docx]

**Supplementary Table 4**. qRT-PCR primer sequences

| **Gene** | **Primer Sequences** |
| --- | --- |
| β-actin | Forward: 5’-AGGGCAGTGATCTCCTTCT-3’ |
|  | Reverse:5’-CAGGGCAGTGATCTCCTTCT-3’ |
| PDCD1 | Forward: 5’-CCCAAGGCGCAGATCAAAGAG-3’ |
|  | Reverse:5’-CAGGACCCAGACTAGCAGC-3’ |
| HAVCR2 | Forward: 5’-AGACAGTGGGATCTACTGCTG-3’ |
|  | Reverse:5’CCTGGTGGTAAGCATCCTTGG-3’ |
| CTLA4 | Forward: 5’-TGGGGAATGAGTTGACCTTC-3’ |
|  | Reverse:5’GCACGGTTCTGGATCAATTA-3’ |
| LAG3 | Forward: 5’-GCTGCATCCTCACCTACAGAG-3’ |
|  | Reverse:5’GCTCCAGCGTACACTGTCAAG-3’ |
| ENTPD1 | Forward: 5’-AGGTGCCTATGGCTGGATTAC-3’ |
|  | Reverse:5’CCAAAGCTCCAAAGGTTTCCT-3’ |
| ICOS | Forward: 5’-ACAACTTGGACCATTCTCATGC-3’ |
|  | Reverse:5’TGCACATCCTATGGGTAACCAG-3’ |
| EOMES | Forward: 5’-CTGCCCACTACAATGTGTTCG-3’ |
|  | Reverse:5’GCGCCTTTGTTATTGGTGAGTT-3’ |
| TNFα | Forward: 5’-CGAGTGACAAGCCTGTAGC-3’ |
|  | Reverse:5’GGTGTGGGTGAGGAGCACAT-3’ |

**Abbreviations：** CTLA4, cytotoxic T-lymphocyte antigen 4; LAG3, lymphocyte activation gene 3; ICOS, inducible costimulator; TNF-α, tumor necrosis factor-α.
