# Supplementary material for: PD1Hi CD8+ T cells correlate with exhausted signature and poor clinical outcome in hepatocellular carcinoma
Source: J Immunother Cancer. 2019 Nov 29;7:331. doi: 10.1186/s40425-019-0814-7 (PMC6884778; doi:10.1186/s40425-019-0814-7)
Supplement: Supplementary file 13 — Additional file 13. Supplementary figure legends. [file 40425_2019_814_MOESM13_ESM.docx]

**Supplementary Figure Legend**

**Figure S1. PD1 expression on HCC infiltrating CD8^+^ T cells and its clinical associations. A and B,** Flow cytometric analysis of the proportions of PD1^-^ (**A**) and PD1^Int^ T cells (**B**) among total CD8^+^ T cells from paired blood, peri-tumor and tumor of HCC patients. **C-F,** Association of patients TNM stages and tumor size with the percentage of CD8^+^PD1^+^ (**C and D**) and CD8^+^PD1^Hi^ among CD8^+^ T cells (**E and F**) from paired blood, peri-tumor and tumor of HCC patient. Error bars indicated median with interquartile rang. Significance was assessed by Wilcoxon matched-pairs signed rank test. *, *P*<0.05; **, *P*<0.01; ***, *P*<0.001; and ****, *P*<0.0001.

**Figure S2. Detection of the mRNA expression levels of exhaustion related markers** **in PD1^Hi^ CD8^+^ TILs.**

The relative mRNA levels of exhaustion related markers including *PDCD1*, *HAVCR2*, *CTLA4*, *LAG3* and *ENTPD1* of CD8^+^PD1^Hi^ and CD8^+^PD1^Int^ TILs were determined by qRT-PCR (n=5). Significance was assessed by Wilcoxon matched-pairs signed rank test. *, *P*<0.05; **, *P*<0.01; ***, *P*<0.001; and ****, *P*<0.0001.

**Figure S3. Expression pattern of transcription factors, apoptotic and proliferative markers of PD1^Hi^ CD8^+^ TILs.**

**A and B,** Representative flow cytometric histograms of various transcription factors (**A**) , apoptotic and proliferative markers (**B**), surface markers (**C**) and cytokine receptors (**D**) on tumor infiltrating PD1^Hi^ (red line), PD1^Int^ (blue line) and PD1^-^ (black line) CD8^+^ TILs. One representative experiment out of three to four was shown.

**Figure S4. Expression pattern of chemokine receptors of PD1^Hi^ CD8^+^ TILs and phenotypic characteristics of TIM3^-^PD1^Hi^ and TIM3^+^PD1^Hi^ TILs.**

**A and B,** Representative flow cytometric histograms of CCR (**A**) and CXCR (**B**) chemokine receptors on tumor infiltrating PD1^Hi^ (red line), PD1^Int^ (blue line) and PD1^-^ (black line) CD8^+^ TILs. One representative experiment out of three to four was shown. **C**, Representative flow cytometric plot of PD1 and TIM3 expression on HCC infiltrating CD8^+^T cells. **D-F**, Expression of co-inhibitory receptors **(D),** exhaustion related transcription factors, apoptotic and proliferative markers **(E)** and activation markers **(F)** on tumor infiltrating PD1^-^, PD1^Int^, TIM3^-^PD1^Hi^ and TIM3^+^PD1^Hi^ CD8^+^T cells. **G**, Representative flow cytometric histograms of cytokines and cytotoxic molecules, including IFN-γ and TNF-α (following the stimulation of PMA, ionomycin and brefeldin A for 5 hours), intracellular Granzyme B and perforin, and CD107a expression (following the overnight stimulation of anti-CD3/CD28) of tumor infiltrating PD1^-^ (black line), PD1^Int^ (blue line), TIM3^-^PD1^Hi^ (orange line) and TIM3^+^PD1^Hi^ (red line) T cells. One representative experiment out of three to four was shown.

**Figure S5. Sorting strategy of PD1^Hi^ CD8^+^ TILs.**

**A and B**, Gating strategy of tumor-infiltrating CD8^+^ T cells based on PD1 and TIM3 expression (**A**) and post sort analysis of PD1-high, PD1-intermediate and PD1-negative CD8^+^ TILs subpopulation (**B**).

**Figure S6. Enriched exhausted PD1^Hi^ CD8^+^ T cells in HCC tumors.**

**A**, Representative IHC images showed the staining for CD3, CD8, PD1 and TIM3 in HCC tumor and peri-tumor. Scale bar, 50μm. **B**, The 5-color multiplex immunofluorescence panel was applied on TMAs. Scale bar, 200μm. **C**, Flow cytometry alike density plot was defining “PD1^Hi^” and “TIM3^+^”-threshold based on quantitative mean pixel fluorescence intensity. **D**, Comparisons of the frequency of CD8^+^PD1^Int^ T cells, CD8^+^PD1^Hi^ T cells, CD8^+^TIM3^-^PD1^Hi^ T cells and CD8^+^TIM3^+^PD1^Hi^ T cells among CD8^+^PD1^+^ T cells between paired peri-tumor and tumor tissues in the validation cohort (n=254). Error bars indicated median with interquartile range. Significance was assessed by Wilcoxon matched-pairs signed rank test. *, *P*<0.05; **, *P*<0.01; ***, *P*<0.001; and ****, *P*<0.0001.

**Figure S7. Prognostic significance of the subsets of CD8^+^ TILs in the validation cohort.**

**A-D,** Kaplan-Meier analysis of overall survival (OS, **A** and **C**) and relapse free survival (RFS, **B** and **D**) in HCC tumors according to the proportion of CD8^+^PD1^Int^, CD8^+^PD1^Hi^ **(A** and **B)**, CD8^+^PD1^+^ TILs, CD8^+^TIM3^-^PD1^Hi^ and CD8^+^TIM3^+^PD1^Hi^ **(C** and **D)** among CD8^+^PD1^+^ TILs in the validation cohort (n=254). **E,** Correlation analysis between the density of CD8^+^TIM3^+^PD1^Hi^, CD8^+^TIM3^-^PD1^Hi^, CD8^+^PD1^Int^ and the density of PDL1^+^ tumor cells (PDL1^+^CD68^-^) per core respectively. Correlation was evaluated by the Spearman correlation coefficient.
